# Gasdermin E mediates resistance of pancreatic adenocarcinoma to enzymatic digestion through a YBX1–mucin pathway

**DOI:** 10.1038/s41556-022-00857-4

**Published:** 2022-03-15

**Authors:** Jiadi Lv, Yuying Liu, Siqi Mo, Yabo Zhou, Fengye Chen, Feiran Cheng, Cong Li, Dilizhatai Saimi, Mengyu Liu, Huafeng Zhang, Ke Tang, Jingwei Ma, Zhenfeng Wang, Qiangqiang Zhu, Wei-Min Tong, Bo Huang

**Affiliations:** 1 Department of Immunology and National Key Laboratory of Medical Molecular Biology, Institute of Basic Medical Sciences, Chinese Academy of Medical Sciences (CAMS) and Peking Union Medical College, Beijing, China; 2 Department of Medical Oncology, National Cancer Center/National Clinical Research Center for Cancer/Cancer Hospital, CAMS and Peking Union Medical College, Beijing, China; 3 Department of Biochemistry and Molecular Biology, Tongji Medical College, Huazhong University of Science and Technology, Wuhan, China; 4 Department of Pathology, Institute of Basic Medical Sciences, CAMS and Peking Union Medical College, Beijing, China

**Keywords:** Pancreatic cancer, Mechanisms of disease, Glycoproteins

## Abstract

Pancreatic ductal adenocarcinoma (PDAC) originates from normal pancreatic ducts where digestive juice is regularly produced. It remains unclear how PDAC can escape autodigestion by digestive enzymes. Here we show that human PDAC tumour cells use gasdermin E (GSDME), a pore-forming protein, to mediate digestive resistance. GSDME facilitates the tumour cells to express mucin 1 and mucin 13, which form a barrier to prevent chymotrypsin-mediated destruction. Inoculation of *GSDME*^−/−^ PDAC cells results in subcutaneous but not orthotopic tumour formation in mice. Inhibition or knockout of mucin 1 or mucin 13 abrogates orthotopic PDAC growth in NOD-SCID mice. Mechanistically, GSDME interacts with and transports YBX1 into the nucleus where YBX1 directly promotes mucin expression. This GSDME–YBX1–mucin axis is also confirmed in patients with PDAC. These findings uncover a unique survival mechanism of PDAC cells in pancreatic microenvironments.

## Abstract (web summary)

Lv et al. reveal a non-canonical role for gasdermin E in protecting pancreatic cancer cells from chymotrypsin-mediated digestion in the microenvironment by promoting the transcription factor YBX1 to induce mucin expression.

## Main

Both tumorigenesis and pathogenesis of pancreatic ductal adenocarcinoma (PDAC) remain incompletely understood. The exocrine portion of the pancreas, the origin of PDAC, constitutes the majority (>95%) of the pancreatic mass, which includes acinar and duct cells and secretes digestive enzymes^1,2^. Physiologically, the secreted pancreatic juice flows through the pancreatic duct into the duodenum and aids digestion. However, this juice is potentially dangerous and is able to destroy neighbouring pancreatic cells under certain conditions such as acute pancreatitis^3,4^. Therefore, normal structures of the pancreatic duct are organized in a strict and orderly manner to avoid self-digestion. During malignant development, it is highly probable that disorderly tumour growth inevitably obstruct the normal ductal space, which leads to pancreatic juice leaking out and destroying nearby cells^5,6^. This raises a fundamental question of how PDAC tumour cells can evade pancreatic enzymatic destruction and survive.

Recent studies have highlighted a pivotal role of the gasdermin family members in mediating inflammatory cell death through their pore-forming activity^7–10^. Gasdermin interdomain cleavage allows the amino-terminal domain to bind membrane phospholipids and oligomerize into a pore on the plasma membrane, which leads to rapid cellular swelling, large bubbles emerging from the plasma membrane and subsequent cell lysis. This gasdermin-mediated programmed necrosis is called pyroptosis. Among the members, gasdermin E (GSDME) is unique because its active form requires cleavage by caspase-3, an enzyme involved in tumour cell apoptosis^11,12^. GSDME is silenced in various tumour cell types due to high methylation of the *GSDME* promoter region^13–15^. However, GSDME is also present in some tumours and can induce tumour cell death^11^ and enhance antitumour immunity^15^. But it is hard to explain why tumour cells would express GSDME to kill themselves. It may be that GSDME plays a tumour-promoting role under certain conditions. In the present study, we show that PDAC tumour cells express GSDME at high levels to mediate resistance to pancreatic enzymatic digestion through a GSDME–YBX1–mucin pathway, thereby playing a tumour-promoting role beyond the known pore-forming function. These findings provide a deeper understanding of the pathogenesis of PDAC with chronic inflammation^16^, which considers the potential enzymatic digestion of paracancerous parenchymal cells.

## Results

### GSDME is required for orthotopic PDAC growth

**Fig. 1:**
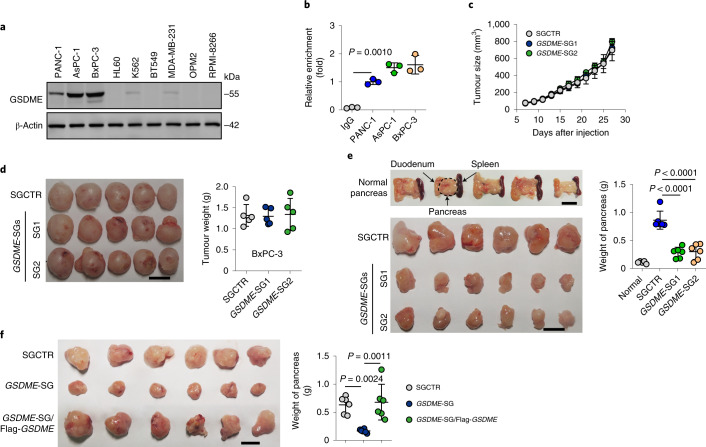
GSDME regulates pancreatic tumour growth. **a**, Western blot analysis of GSDME expression in PANC-1, AsPC-1, BxPC-3, HL60, K562, BT549, MDA-MB-231, OPM2 and RPMI-8266 cells. **b**, ChIP–qPCR analysis performed with anti-5hMC and primers specific for *GSDME* in PANC-1, AsPC-1 and BxPC-3 cells. **c**,**d**, BxPC-3 cells transfected with SGCTR or *GSDME*-SGs (2 × 10^6^ cells) were subcutaneously injected into mice. Tumours were sized (**c**, *n* = 6), photographed and weighed (**d**, *n* = 5). **e**,**f**, BxPC-3 cells transfected with SGCTR, *GSDME*-SGs or *GSDME*-SG/Flag-*GSDME* (2.5 × 10^5^ cells) were orthotopically injected into the pancreas of mice. Tumours were photographed (left) and weighed (right) (*n* = 6). Normal pancreas served as the control. Scale bars, 1 cm. In **a** and **b**, *n* = 3 biological independent experiments. *P* values were determined by one-way ANOVA Bonferroni’s test (**b**–**f**). The data represent the mean ± s.d. Source data

**Extended Data Fig. 1:**
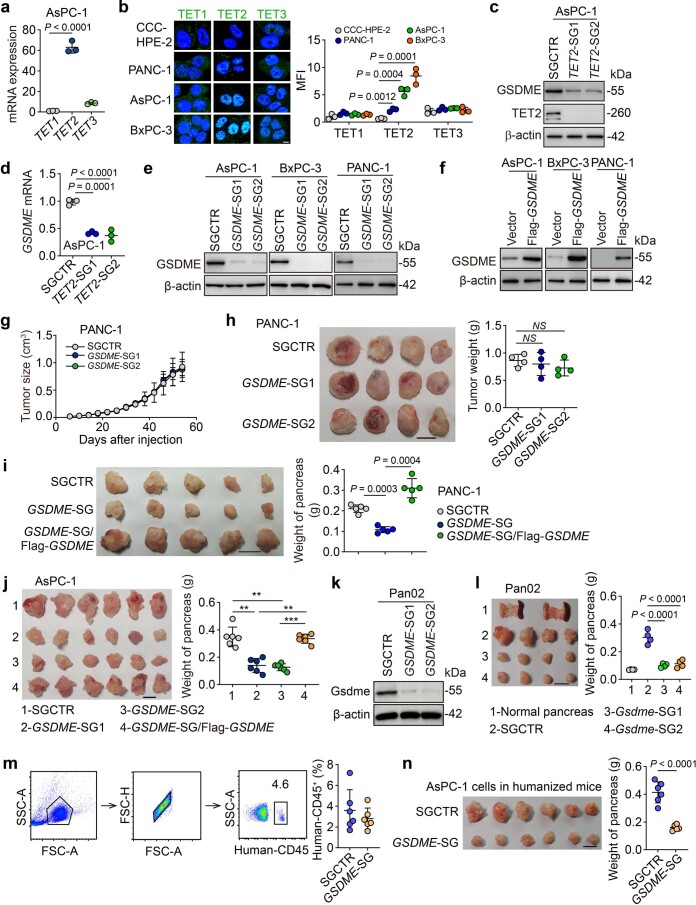
GSDME facilitated pancreatic tumor growth. **a**, The expression of *TET1-3* from AsPC-1 cells was determined by real-time PCR. **b**, TET1, TET2, TET3 (green color) and DAPI immunostaining in CCC-HPE-2, PANC-1, AsPC-1 and BxPC-3 cells. The mean fluorescence intensity (MFI) of TET1-3 was calculated by Image J. Scale bar, 5 μm. **c**, Western blot analysis of GSDME and TET2 expression from SGCTR and *TET2*-SGs- PANC-1 or AsPC-1 cells. **d**, The expression of GSDME in SGCTR or *TET2*-SGs was detected by real-time PCR. **e**, The knockout efficiency of GSDME in PANC-1, AsPC-1 or BxPC-3 cells was determined by western blot. **f**, The expression of GSDME in vector or Flag-GSDME-overexpressing PANC-1, AsPC-1 or BxPC-3 were determined by western blot. **g**,**h**, SGCTR or *GSDME*-SGs- PANC-1 cells (2 × 10^6^) were subcutaneously injected into mice. Tumor growth was measured (g, n = 6/group). Tumors were presented photographically (h, left, n = 4/group) or weighed (h, right). **i**,**j**, SGCTR, *GSDME*-SGs or *GSDME*-SG/Flag-*GSDME*-PANC-1 cells (i, 5 × 10^5^ cells) or AsPC-1 cells (j, 2.5 × 10^5^ cells) were orthotopically injected into mice. Tumors were presented photographically (left) or weighed (right) (i, n = 5/group; j, n = 6/group). **k**, The knockout efficiency of *Gsdme* in Pan02 cells was determined by western blot. **l**, SGCTR or *Gsdme*-SGs-Pan02 cells (1 × 10^6^ cells) were orthotopic injected into the pancreas of C57BL/6 mice. 40 days after injection, tumors were presented photographically (left) and weighted (right) (n = 4/group). The normal pancreas was served as control. Scale bar, 1 cm. **m**, The peripheral lymphocytes from humanized mice were analyzed by FACS using an anti-human CD45 antibody before the subsequent animal experiments (n = 6). **n**, SGCTR and *GSDME*-SG-AsPC-1 cells (2.5 × 10^5^ cells) were orthotopic injected into the pancreas of humanized mice. Tumors were presented photographically (left) and weighted (right) (n = 6/group). Scale bar, 1 cm. In **a-c** and **f**, n = 3 biological independent experiments. **P < 0.01, ***P < 0.001, by two-tailed one-way ANOVA Bonferroni’s test (**a**,**d**,**h-j**, **l** and **n**) or two-tailed student’s t-test (**b**). The data represent mean ± SD. Source data

GSDME was expressed at high levels in human PDAC tumour cell lines (Fig. 1a). In line with this, the *GSDME* promoter region was highly hydroxymethylated (Fig. 1b), and ten-eleven-translocation methylcytosine dioxygenase 2 (TET2) was strongly upregulated in PDAC cell lines and primarily found in the nucleus (Extended Data Fig. 1a,b). Knocking down *TET2* downregulated GSDME expression in PDAC cells (Extended Data Fig. 1c,d), which suggests that PDAC cells use an altered epigenetic programme to express GSDME at high levels. Such a pattern of GSDME expression prompted us to explore the role of GSDME in PDAC cells by using the CRISPR–Cas9 technique to knockout *GSDME*. We subcutaneously or orthotopically injected BxPC-3 cells transfected with single guide RNAs (sgRNAs) targeting *GSDME* (*GSDME*-SGs) into NOD-SCID mice. GSDME deficiency did not affect subcutaneous tumour growth (Fig. 1c,d and Extended Data Fig. 1e), whereas orthotopic tumour growth was markedly inhibited in the pancreas (Fig. 1e). This phenomenon was not due to local immune surveillance because immunodeficient mice were used. We speculated that the pancreas probably produces certain factors that are toxic to these GSDME knockout cells, thus disrupting their growth. Indeed, re-expressing GSDME conferred the ability of BxPC-3 cells transfected with *GSDME*-SGs to grow a tumour in the pancreas (Fig. 1f and Extended Data Fig. 1f). Similar results were obtained in PANC-1 and AsPC-1 cells that had *GSDME* knocked out. These cells rapidly formed a subcutaneous tumour but exhibited weak tumorigenicity in the pancreas, but GSDME re-expression rescued orthotopic tumour growth (Extended Data Fig. 1g–j). Given the role of GSDME in tumour immunomodulation^15^, we used immunocompetent mouse models to verify this by constructing *GSDME* knockout mouse Pan02 cells and human AsPC-1 cells and orthotopically inoculating them into wild-type (WT) C57BL/6J and humanized mice, respectively. GSDME deficiency in these models similarly suppressed tumour growth (Extended Data Fig. 1k–n). Together, these results suggest that GSDME is required for the orthotopic growth of pancreatic tumours.

### GSDME mediates resistance of PDAC cells to digestive enzymes

**Fig. 2:**
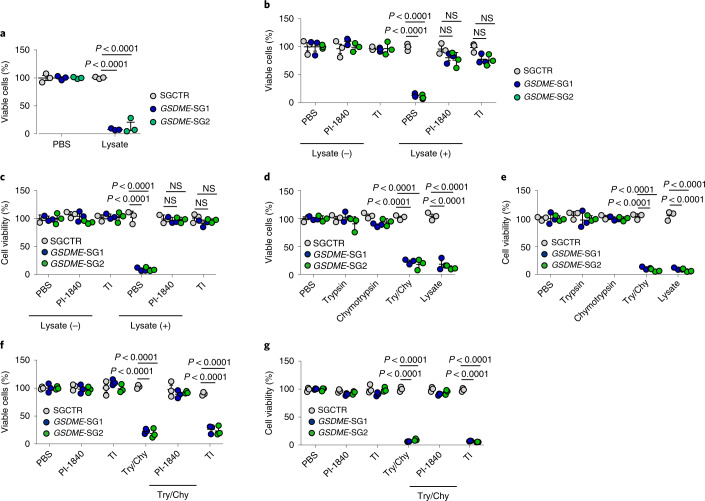
GSDME helps tumour cells to resist trypsin and chymotrypsin digestion. **a**, AsPC-1 cells transfected with SGCTR or *GSDME*-SGs were treated with lysate (20 μl ml^–1^) isolated from mouse pancreas for 72 h. Viable cells were determined by trypan blue (TB) staining. **b**,**c**, AsPC-1 cells transfected with SGCTR or *GSDME*-SGs were treated with or without lysate (20 μl ml^–1^) in the presence of PI-1840 (100 μM) or a trypsin inhibitor (TI; 50 μg ml^–1^) for 72 h. Cell viability was measured by TB staining (**b**) or an ATP cell viability assay (**c**). **d**,**e**, AsPC-1 cells transfected with SGCTR or *GSDME*-SGs were treated with PBS, trypsin (50 U ml^–1^), chymotrypsin (40 U ml^–1^), trypsin and chymotrypsin (Try/Chy) or lysate for 72 h. Cell viability was determined by TB staining (**d**) or an ATP cell viability assay (**e**). **f**,**g**, The same as **b**–**e**, except that some cells were pretreated with Try/Chy for 10 min and then treated with IP-1840 or a trypsin inhibitor for 72 h. For **a**–**g**, *n* = 3 biological independent experiments. *P* values were determined by one-way ANOVA Bonferroni’s test (**a**–**g**). NS, not significant. The data represent the mean ± s.d. Source data

**Extended Data Fig. 2:**
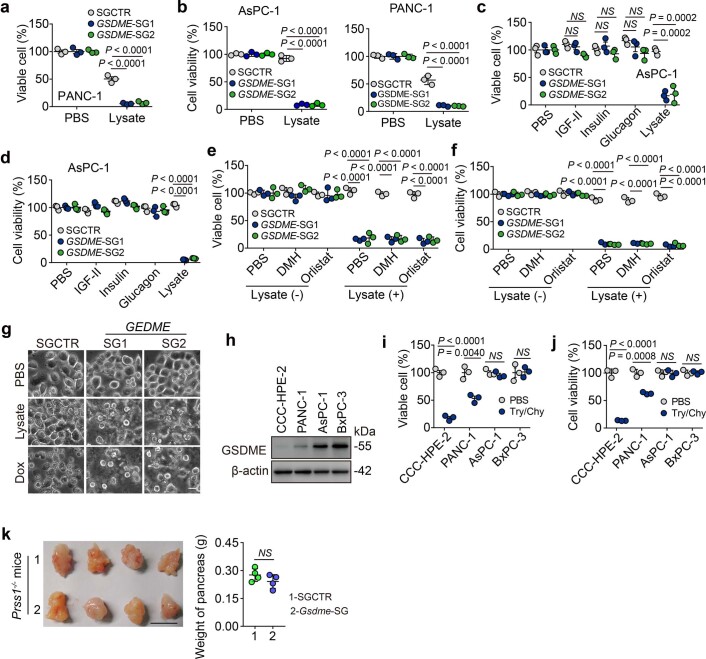
GSDME protected tumor cells from enzymatic digestion. **a**,**b**, SGCTR or *GSDME*-SGs- AsPC-1 or PANC-1 cells were stimulated with lysate (20 μl/ml) for 72 hr. The viable cells were measured by TB staining (a) or ATP Cell Viability Assay (b). **c**,**d**, The same as (a), except that the AsPC-1 cells were treated with IGF-II (2 ng/ml), Insulin (1 μg/ml), glucagon (0.5 ng/ml) or lysate. Cell viability was detected by TB staining (c) or ATP cell viability assay (d). **e**,**f**, SGCTR or *GSDME*-SGs- AsPC-1 cells were treated with lysate, DMH (10 μM), Orlistat (12.5 μM), lysate + DMH or lysate + Orlistat for 72 hr. Cell viability was determined by TB staining (e) or ATP cell viability assay (f). **g**, SGCTR, *YBX1*-SGs-AsPC-1 cells were treated with PBS, Try/Chy or Doxorubicin (50 μM) for 48 hr. The representative images were shown. Scale bar, 20μm. **h**, The expression of GSDME from CCC-HPE-2, PANC-1, AsPC-1, and BxPC-3 were analyzed by western blot. **i**,**j**, CCC-HPE-2, PANC-1, AsPC-1 or BxPC-3 cells were treated with trypsin/chymotrypsin (Try/Chy) for 72 hr. Cell viability was determined by TB staining (i) or ATP cell viability assay (j). **k**, SGCTR and *GSDME*-SG-Pan02 cells (1 × 10^6^ cells) were orthotopic injected into the pancreas of *Prss1*^−/−^ C57BL/6JGpt mice. 40 days after injection, tumors were presented photographically (left) and weighted (right) (n = 4). Scale bar, 1 cm. In a-f, i and j, n = 3 biological independent experiments. NS, no significance. **P < 0.01, ***P < 0.001, by one-way ANOVA Bonferroni’s test (**a-f**) or student’s t-test (**i**,**j** and **k**). The data represent mean ± SD. Source data

Next, we explored whether GSDME mediates PDAC resistance to pancreatic enzymatic digestion, thus explaining the tumour suppression induced by GSDME deficiency. To test this, we used pancreatic lysates to treat PDAC cells. GSDME deficiency markedly decreased the viability of PDAC cells (Fig. 2a and Extended Data Fig. 2a,b). Insulin-like growth factor 2, insulin and glucagon are not involved in *GSDME*-SG-mediated tumour cell death in vitro (Extended Data Fig. 2c,d); therefore we speculated that exocrine enzymes are involved in GSDME-deficient tumour cell death. Pancreatic exocrine enzymes are composed of trypsin, chymotrypsin, amylase and lipase. The addition of an amylase or a lipase inhibitor did not affect pancreatic-lysate-mediated tumour cell death (Extended Data Fig. 2e,f); however, cell death was blocked by either a chymotrypsin or a trypsin inhibitor (Fig. 2b,c). This result suggests that trypsin and/or chymotrypsin in the pancreatic digestive juice is involved in *GSDME*^−/−^ PDAC cell death. To further confirm this result, we used trypsin and chymotrypsin to treat cells transfected with *GSDME*-SGs. Trypsin or chymotrypsin alone did not cause cell death in cells transfected with *GSDME*-SGs, but the combination of these two enzymes did (Fig. 2d,e). As chymotrypsin is produced as an inactive form in the pancreas and activated by trypsin, we treated cells with both trypsin and chymotrypsin and then added a trypsin inhibitor or a chymotrypsin inhibitor to the medium. Only the chymotrypsin inhibitor blocked cell death (Fig. 2f,g), which suggests that chymotrypsin exerts a direct cytotoxic effect on *GSDME*-SGs pancreatic tumour cells. Although previous reports have indicated that GSDME mediates pyroptosis, in this study, we found that pancreatic lysate did not induce pyroptosis of cells (Extended Data Fig. 2g). In addition, unlike malignant pancreatic cells, normal human pancreatic cells, which express GSDME at low levels (Extended Data Fig. 2h), were not resistant to trypsin or chymotrypsin (Extended Data Fig. 2i,j). Next, we used *Prss1*^−/−^ C57BL/6J mice (in which trypsinogen is knocked out) to further validate these in vitro results in vivo. Trypsin deficiency led to *GSDME*^–/–^ Pan02 cells to grow in the pancreas, similar to the control cells (Extended Data Fig. 2k). Together, these results suggest that pancreatic tumour cells mobilize GSDME to resist exocrine digestive enzymes.

### GSDME induces mucins for resistance to digestive enzymes

**Fig. 3:**
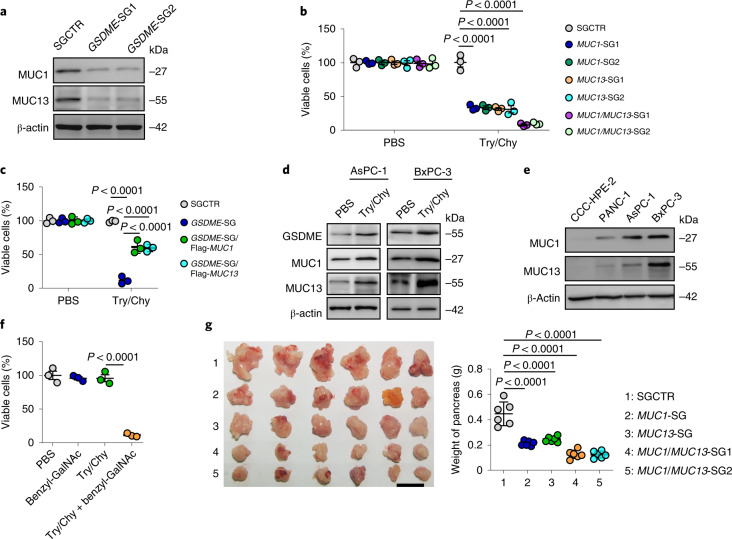
MUC1 and MUC13 mediate tumour growth. **a**, The expression of MUC1 and MUC13 from AsPC-1 cells transfected with SGCTR or *GSDME*-SGs was determined by western blotting. **b**, AsPC-1 cells transfected with SGCTR, *MUC1*-SGs, *MUC13*-SGs or *MUC1/MUC13*-SGs were treated with PBS or Try/Chy for 72 h. Viable cells were measured by TB staining. **c**, The same as **b**, except that AsPC-1 cells transfected with *GSDME*-SG, *GSDME*-SG/Flag-*MUC1* or *GSDME*-SG/Flag-*MUC13* were used. **d**, AsPC-1 or BxPC-3 cells were treated with PBS or Try/Chy for 48 h. The expression levels of GSDME, MUC1 and MUC13 were analysed by western blotting. **e**, The same as **d**, except that CCC-HPE-2, PANC-1, AsPC-1 and BxPC-3 cells were used. **f**, The same as **b**, except that AsPC-1 cells were treated with benzyl-GalNAc (2 mM), Try/Chy or Try/Chy plus benzyl-GalNAc. **g**, AsPC-1 cells transfected with SGCTR, *MUC1*-SGs, *MUC13*-SGs or *MUC1/MUC13*-SGs (2.5 × 10^5^ cells) were orthotopically injected into the pancreas of mice. Tumours were photographed (left) and weighed (right) (*n* = 6 per group). Scale bar, 1 cm. For **a**–**f**, *n* = 3 biological independent experiments. *P* values were determined by one-way ANOVA Bonferroni’s test (**b**,**c**,**f**,**g**). The data represent the mean ± s.d. Source data

**Extended Data Fig. 3:**
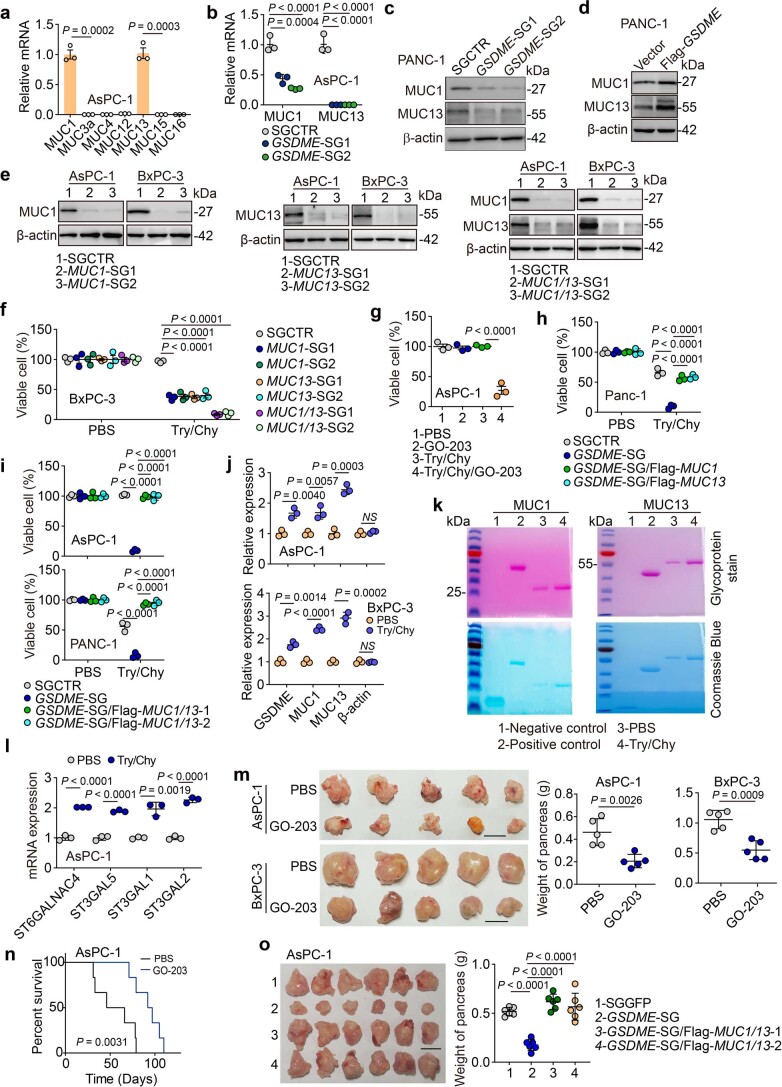
MUC1/13 was identified as the protectors of tumor cells against enzymatic digestion. **a**, The mRNA expression of *MUCs* (1, 3a, 4, 12, 13, 15 and 16) from AsPC-1 cells was determined by real-time PCR. **b**, The mRNA expression of *MUC1* and *MUC13* from SGCTR or *GSDME*-SGs-AsPC-1 cells was determined by qPCR. **c**, The expression of MUC1 and MUC13 from SGCTR or *GSDME*-SGs- PANC-1 cells was determined by western blot. **d**, The level of MUC1 and MUC13 from vector or Flag-*GSDME*-PANC-1 cells was determined by western blot. **e** The knockout efficiency of MUC1, MUC13 or MUC1/13 from AsPC-1 or BxPC-3 cells was determined by western blot. **f**,**i**, SGCTR, *MUC1*-SGs, *MUC13*-SGs or *MUC1/13*-SGs-BxPC-3 cells (f) or SGCTR, *GSDME*-SG or *GSDME*-SG/Flag- *MUC1*/*13*-AsPC-1 or PANC-1 cells (i) were treated with Trp/Chy for 72 hr. The viable cells were counted by TB staining. **g**, AsPC-1 cells were treated with GO-203 (5 μM), trypsin (0.5 mg/ml) /chymotrypsin (1 mg/ml) or trypsin/chymotrypsin/GO203 for 72 hr. The viable cells were counted by TB staining. **h**, *GSDME*-SG, *GSDME*-SG/Flag-*MUC1* and *GSDME*-SG/Flag-*MUC13*- PANC-1 cells were treated with PBS or Try/Chy for 72 hr. The viable cells were measured by TB staining. **j**, AsPC-1 or BxCP-3 cells were treated with PBS or Try/Chy for 48 hr. The level of GSDME, MUC1, MUC13 and β-actin was determined by analyzing the gray value on the western blot protein bands. **k**, AsPC-1 cells were treated with Try/Chy for 48 hr. Glycoprotein staining of MUC1 and MUC13 pulled down by immunoprecipitation assay (top). Coomassie blue staining panel represents the total amount of MUC1/13 (bottom). **l**, The mRNA expression of *ST6GalNaC4*, *ST3Gal5*, *ST3Gal1* or *ST3Gal2* from AsPC-1 cells treated with Trp/Chy for 24 hr. **m**,**n**, AsPC-1 or BxPC-3 cells (2.5 × 10^5^ cells) were orthotopically injected into mice. After 3 days of inoculation, mice were treated with GO-203 (14 mg/kg, i.p.) once every two days for 20 days. Tumors were presented photographically (m, left) or weighed (m, right) (n = 5/group) and the mouse survival was recorded (n) (n = 6/group). Scale bar, 1 cm. **o**, SGCTR, *GSDME*-SG, *GSDME*-SG/Flag*-Muc1/13*-1 or *GSDME*-SG/Flag*- Muc1/13*-2 -AsPC-1 cells (2.5 × 10^5^ cells) were orthotopic injected into the pancreas of NSG mice. 30 days after injection, tumors were presented photographically (left) and weighted (right) (n = 6/group). Scale bar, 1 cm. In **a**-**j** and **l**, n = 3 biological independent experiments. NS, no significance. NS, no significance. *P < 0.05, **P < 0.01, ***P < 0.001, by one-way ANOVA Bonferroni’s test (**b**,**f-j** and **o**), two-tailed Student’s t-test (**a** and **l**) or the Log-rank survival analysis (**n**). The data represent mean ± SD. Source data

Next, we investigated the molecular mechanism by which GSDME regulates the resistance to digestive enzymes. The mucosal epithelium can protect itself by expressing a layer of mucous molecules to retain particles and exogenous enzymes, which prompted us to hypothesize that GSDME promotes resistance to digestive enzymes by upregulating mucin expression. In line with this hypothesis, measurement of the membrane-associated mucins MUC1, MUC3, MUC4, MUC12, MUC13, MUC15 and MUC16 (ref. ^17^) showed that MUC1 and MUC13 are expressed at high levels in pancreatic tumour cells (Extended Data Fig. 3a). Moreover, knocking out *GSDME* in AsPC-1 or PANC-1 cells resulted in the downregulation of MUC1 and MUC13 (Fig. 3a and Extended Data Fig. 3b,c), and *GSDME* overexpression upregulated MUC1 and MUC13 (Extended Data Fig. 3d). Knockout or inhibition of mucin decreased the resistance of PDAC cells to digestive enzymes (Fig. 3b and Extended Data Fig. 3e–g). In addition, although *GSDME*^−/−^ tumour cells lost the resistance to digestive enzymes, re-expression of exogenous MUC1 or MUC13 largely restored their resistance (Fig. 3c and Extended Data Fig. 3h), and the co-expression of MUC1 and MUC13 achieved the greatest resistance (Extended Data Fig. 3i). Meanwhile, trypsin and chymotrypsin treatment upregulated the expression of GSDME, MUC1 and MUC13 (Fig. 3d and Extended Data Fig. 3j). In contrast, normal human pancreatic cells expressed markedly low levels of MUC1 and MUC13 (Fig. 3e) concomitant with the loss of resistance to digestive enzymes (Extended Data Fig. 2j,k). The carbohydrate chains on mucins are commonly initiated with *N*-acetylgalactosamine (GalNAc) by linking to a mucin serine or threonine hydroxyl group. This O-linked glycosylation of MUC1 and MUC3 was observed in PDAC cells (Extended Data Fig. 3k), which is consistent with previous reports^18,19^. Moreover, trypsin and chymotrypsin treatment promoted such glycosylation (Extended Data Fig. 3k), whereas blocking the glycosylation of mucins by the addition of benzyl-GalNAc, an inhibitor of *N*-acetylgalactosaminyltransferase, disrupted the resistance to trypsin and chymotrypsin (Fig. 3f). In line with this result, mRNA expression of sialyltransferases (*ST6GALNAC4*, *ST3GAL1*, *ST3GAL2* and *ST3GAL5*) was upregulated by the addition of trypsin and chymotrypsin (Extended Data Fig. 3l). To validate these results in vivo, *MUC1*-SG, *MUC13*-SG or *MUC1*/*MUC13*-SG tumour cells were inoculated into the pancreas of mice. Knockout of either MUC1 or MUC13 retarded tumour growth, but the knockout of both achieved the greatest tumour regression (Fig. 3g). Similarly, the use of a mucin inhibitor inhibited tumour growth and prolonged the survival of mice (Extended Data Fig. 3m,n). In addition, overexpression of MUC1 or MUC13 rescued GSDME-deficiency-retarded tumour growth (Extended Data Fig. 3o). Together, these results suggest that GSDME induces the expression of MUC1 and MUC13, which in turn promotes the resistance of PDAC cells to digestive enzymes.

### GSDME interacts with YBX1 to express mucin

**Fig. 4:**
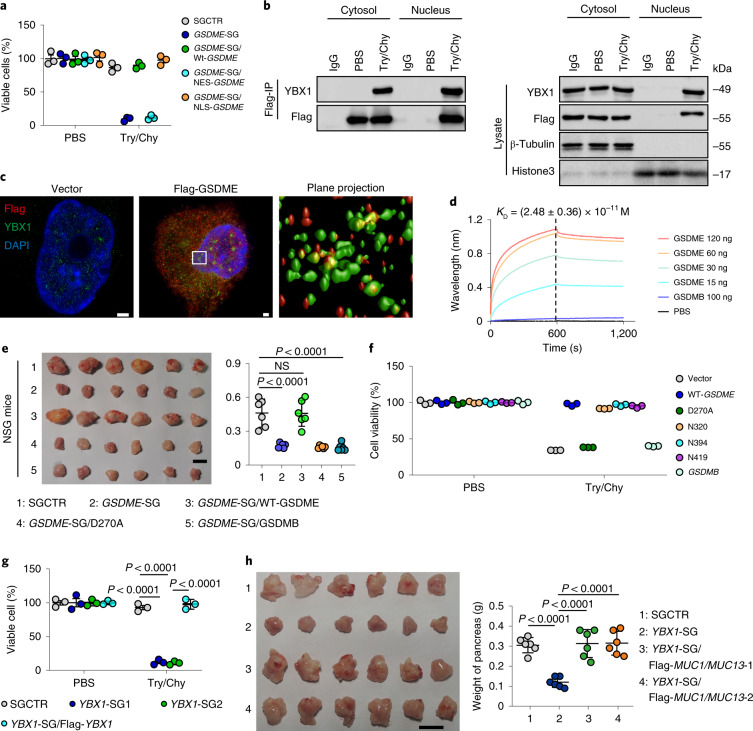
GSDME binds YBX1 and translocates into the nucleus. **a**, AsPC-1 cells transfected with SGCTR, *GSDME*-SG, *GSDME*-SG/WT-*GSDME, GSDME*-SG/NES-*GSDME* or *GSDME*-SG/NLS-*GSDME* were treated with Try/Chy for 72 h. Viable cells were measured by TB staining. **b**, AsPC-1 cells were treated with Try/Chy for 36 h. The cytosolic or nuclear fraction was extracted to perform an immunoprecipitation (IP) assay with anti-Flag-GSDME antibody (left). The expression of YBX1 was analysed by western blotting (right). **c**, AsPC-1 cells transfected with *GSDME*-SG/Vector or *GSDME*-SG/Flag-GSDME were treated with Try/Chy for 24 h. The cells were stained with anti-Flag and YBX1 antibodies and observed under a Stedycon super-resolution microscope. The plane projection of the Flag-GSDME AsPC-1 cell is indicated on the right, and the yellow spots represent colocalization of Flag-GSDME and YBX1. Scale bar, 1 μm. **d**, Binding (*K*_D_) between GSDME and YBX1 was measured by bio-layer interferometry. GSDMB was used as a negative control. **e**, AsPC-1 cells transfected with SGCTR, *GSDME*-SG, *GSDME*-SG/WT-*GSDME*, *GSDME*-SG/D270A or *GSDME*-SG/*GSDMB* (2.5 × 10^5^ cells) were orthotopically injected into the pancreas of NSG mice. At 30 days after injection, tumours were photographed (left) and weighed (right) (*n* = 6 per group). Scale bar, 1 cm. **f**, AsPC-1 cells transfected with *GSDME*-SG/Vector, *GSDME*-SG/WT-*GSDME*, *GSDME*-SG/D270A, *GSDME*-SG/N320, *GSDME*-SG/N394, *GSDME*-SG/N419 or *GSDME*-SG/*GSDMB* were treated with PBS or Try/Chy for 48 h. Cell viability was measured. **g**, The same as **a**, except that AsPC-1 cells transfected with SGCTR, *YBX1*-SGs or *YBX1*-SG/Flag-*YBX1* were used. **h**, AsPC-1 cells transfected with SGCTR, *YBX1*-SG or *YBX1*-SG/Flag-*MUC1/MUC13* (2.5 × 10^5^ cells) were orthotopically injected into the pancreas of mice. Tumours were photographed (left) and weighed (right) (*n* = 6 per group). Scale bar, 1 cm. For **a**–**d**, **f** and **h**, *n* = 3 biological independent experiments. *P* values were determined by one-way ANOVA Bonferroni’s test (**a**,**e**–**h**). The data represent the mean ± s.d. Source data

**Extended Data Fig. 4:**
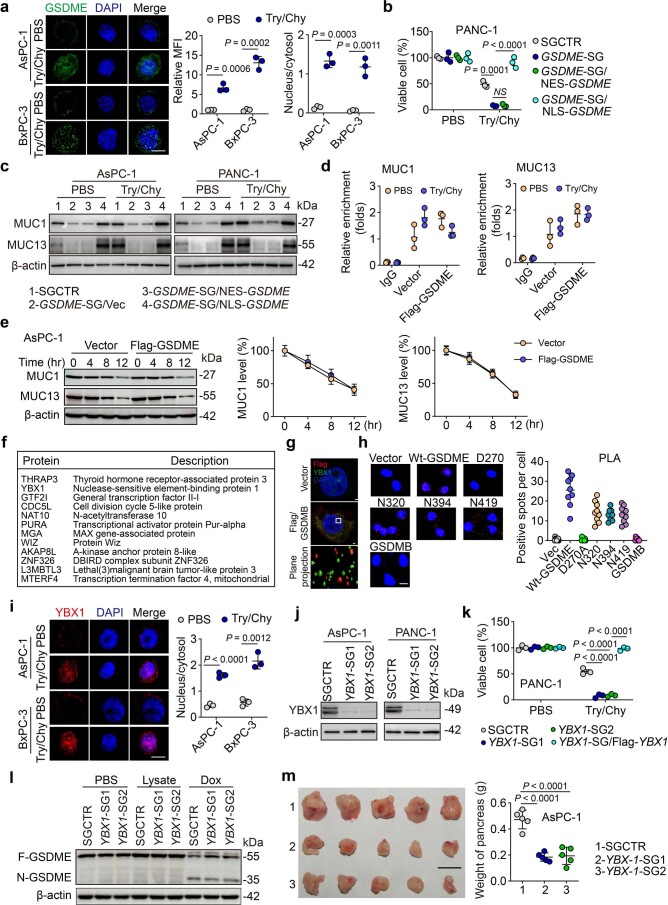
GSDME-YBX1-MUCs pathway regulated tumor growth. **a**, Immunostaining of GSDME in Trp/Chy–treated AsPC-1 and BxPC-3 cells. The mean fluorescence intensity (MFI) or fluorescence intensity of nucleus/cytosol was calculated by Image J. Scale bars, 10 μm. **b**, SGCTR, *GSDME*-SG, *GSDME*-SG/NES-*GSDME* and *GSDME*-SG/NLS-*GSDME*-PANC-1 cells were treated with Try/Chy for 72 hr. The viable cells were measured by TB staining. **c**, SGCTR, *GSDME*-SG, *GSDME*-SG/NES-*GSDME* or *GSDME*-SG/NLS- *GSDME*-AsPC-1 or PANC-1 cells were treated with Try/Chy for 48 hr. The expression of MUC1 and MUC13 were analyzed by western blot. **d**, Vector and Flag-GSDME-AsPC-1 cells were treated with PBS or Try/Chy for 48 hr. RNA immunoprecipitation assays were performed using an antibody against Flag and relative precipitated RNA of *MUC1* (left) and *MUC13* (right) levels were normalized. Antibody against IgG served as the negative control. **e**, Vector or Flag-GSDME-AsPC-1 cells were treated with Try/Chy for 24 hr, followed by the addition of cycloheximide (20 μM) for indicated time (0, 4, 8 or 12 hr). Cells were lysed to perform western blot analysis with an anti-Muc1 or anti-Muc13 antibody (left). The level of Muc1 (middle) or Muc13 (right) was quantified. **f**, *GSDME*-SG/Flag-*GSDME*-AsPC-1 cells were treated with Try/Chy for 48 hr. Cell lysates were immunoprecipitated with anti-Flag for mass spectrometry. Identified transcriptional regulatory proteins were listed. **g**, *GSDME*-SG/Vector or *GSDME*-SG/Flag-GSDMB-AsPC-1 cells were treated with Try/Chy for 24 hr. The cells were stained with anti-Flag and YBX1 antibodies and observed under the STEDYCON super-resolution microscope. Scale bar, 1 μm. **h**, Representative images of PLA in *GSDME*-SG/Vector, *GSDME*-SG/Wt-*GSDME*, *GSDME*-SG/D270A, *GSDME*-SG/N320, *GSDME*-SG/N394, *GSDME*-SG/N419 or *GSDME*-SG/*GSDMB*-AsPC-1 cells were treated with PBS or Try/Chy for 48 hr. The red spots are regions of signal amplification denoting GSDME and YBX1 interaction. The PLA signals were quantified and analyzed. Scale bars, 10 μm. **i**, The same as (a), except that cells were stained with anti-YBX1 antibody. Scale bars, 10 μm. **j**, The knockout efficiency of YBX1 in AsPC-1 or PANC-1 cells was determined by western blot. **k**, The same as (b), except that SGCTR, *YBX1*-SGs and *YBX1*-SG/Flag-*YBX1*- PANC-1 cells were used. **l**, SGCTR, *YBX1*-SGs-AsPC-1 cells were treated with PBS, Try/Chy or Doxorubicin (50 μM) for 48 hr. The expression of GSDME was analyzed by Western blot. **m**, SGCTR or *YBX1*-SGs-AsPC-1 cells (2.5 × 10^5^ cells) were orthotopically injected into mice. Tumors were presented photographically (left) or weighed (right) (n = 5/group). Scale bars, 1 cm. In **a**-**e** and **h**, n = 3 biological independent experiments. **P < 0.01, ***P < 0.001, by two-tailed Student’s t-test (**a**,**i**) or one-way ANOVA Bonferroni’s test (**b,k** and **m**). The data represent mean ± SD. Source data

Next, we explored the molecular pathway that GSDME uses to regulate MUC1 and MUC13. GSDME is typically found in the cytoplasm where it is cleaved by caspase-3 to exert a pore-forming function. Notably, GSDME was localized in the nucleus in pancreatic tumour cells, which was enhanced by the presence of trypsin and chymotrypsin (Extended Data Fig. 4a). To verify whether nuclear GSDME is involved in the resistance of pancreatic tumour cells to digestive enzymes, cells transfected with *GSDME*-SGs were forced to express either nuclear localization sequence (NLS)-GSDME or nuclear export sequence (NES)-GSDME. NLS-GSDME restored the resistance of cells trasnfected with *GSDME*-SGs to trypsin and chymotrypsin, whereas NES-GSDME had no such effect (Fig. 4a and Extended Data Fig. 4b). Consistently, only NLS-GSDME induced the upregulation of MUC1 and MUC13 (Extended Data Fig. 4c). Such mucin upregulation could not be attributable to the binding of GSDME to *MUC1* or *MUC13* mRNA (Extended Data Fig. 4d) or to GSDME-mediated mucin stabilization (Extended Data Fig. 4e). On the basis of these results, we speculated that unlike the pore-forming effect in the cytosol, GSDME in the nucleus acts as a transcriptional regulator. Following the pull-down of a Flag–GSDME fusion protein, the immunoprecipitants were analysed by mass spectrometry. Although more than 1,000 GSDME-binding proteins were detected in the PDAC tumour cells, among the transcriptional regulatory proteins, transcription factor Y-box-binding protein 1 (YBX1) drew our attention (Extended Data Fig. 4f and Supplementary Table 1). As the most prominent member of the YBX family, YBX1 has been associated with multiple cancer-related processes^20^. Co-immunoprecipitation of the nuclear fraction showed that GSDME bound to YBX1 (Fig. 4b), and ultra-high super-resolution microscopy showed that GSDME colocalized with YBX1 in the nucleus of AsPC-1 cells in which endogenous *GSDME* had been deleted (Fig. 4c). GSDMB, which has similar molecular weight and isoelectric point to GSDME^21,22^, did not colocalize with YBX1 in the nucleus (Extended Data Fig. 4g), which suggests that binding of GSDME to YBX1 is specific. A bio-layer interferometry assay further confirmed a direct interaction between GSDME and YBX1 (Fig. 4d). The aspartate residue 270 (D270) of human GSDME has been identified as the cleavage site for caspase-3 (ref. ^11^). Mutating this aspartate to alanine (D270A) resulted in the disruption of the binding of GSDME to YBX1 (Extended Data Fig. 4h); however, GSDME N-terminal fragments (N320, N394 and N419) did not lose the binding ability, as evidenced by proximal ligation assay results (Extended Data Fig. 4h). In addition, GSDMB could not bind YBX1 (Extended Data Fig. 4h). Using AsPC-1 cells in which *GSDME* was knocked out, we further demonstrated that only WT GSDME (not GSDME-D270A or WT GSDMB) rescued the GSDME-deficiency-retarded tumour growth in the pancreas (Fig. 4e). In line with this result, the in vitro assay showed that neither GSDME-D270A nor GSDMB was able to rescue the resistance of *GSDME* knockout AsPC-1 cells to enzymatic digestion (Fig. 4f). Meanwhile, YBX1 is expressed in PDAC cells (Extended Data Fig. 4i), and *YBX1* knockout resulted in the loss of the resistance to trypsin and chymotrypsin, which could be rescued by the re-expression of YBX1 (Fig. 4g and Extended Data Fig. 4j,k). In addition, although *YBX1* knockout induced PDAC cell death, it did not induce GSDME cleavage following pancreatic lysate treatment (Extended Data Fig. 4l). Notably, *YBX1*^−/−^ PDAC cells barely grew a visible tumour in the pancreas of mice (Extended Data Fig. 4m). Furthermore, exogenous expression of MUC1 and MUC13 rescued the ability of *YBX1*^−/−^ PDAC cells to grow a tumour in the pancreas (Fig. 4h). Together, these results suggest that GSDME regulates MUC1 and MUC13 expression by interacting with the transcription factor YBX1.

### GSDME transports YBX1 into the nucleus for mucin expression

**Fig. 5:**
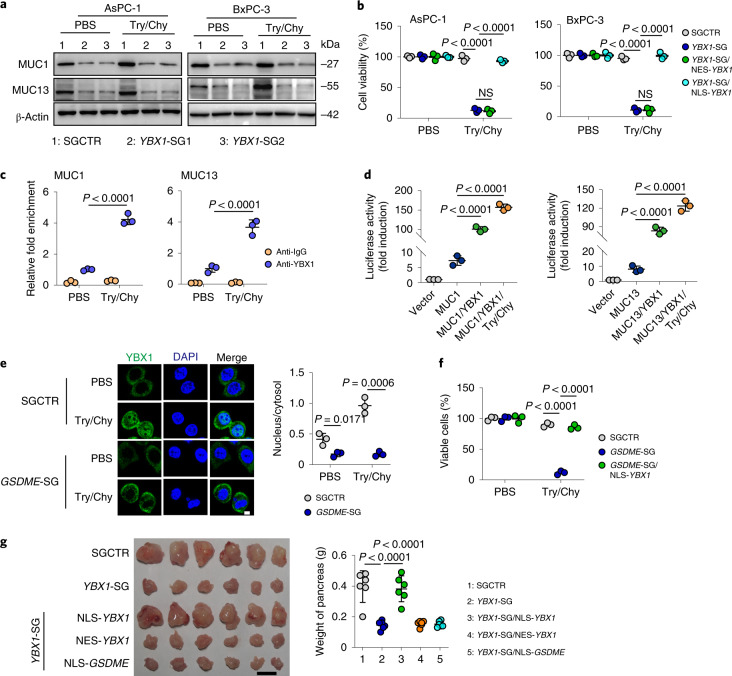
YBX1 binds to the promoters of *MUC1* and *MUC13* to upregulate their expression. **a**, AsPC-1 cells and BxPC-3 cells transfected with SGCTR or *YBX1*-SGs were treated with Try/Chy for 48 h. The expression of MUC1 and MUC13 was analysed by western blotting. **b**, AsPC-1 cells and BxPC-3 cells transfected with SGCTR, *YBX1*-SGs, *YBX1*-SG/NES-*YBX1* or *YBX1*-SG/NLS-*YBX1* were treated with Try/Chy for 72 h. Viable cells were measured by TB staining. **c**, AsPC-1 cells treated with Try/Chy for 24 h were collected for ChIP–qPCR assay with anti-YBX1 and specific primers for MUC1 (left) or MUC13 (right). **d**, HEK-293T cells were co-transfected with MUC1 (left) or MUC13 (right) promoter luciferase reporter PGL3 and YBX1 plasmid for 24 h. Cells were then treated with Try/Chy for another 24 h, followed by analysis of luciferase activity. **e**, Immunostaining images (left) and quantification (right) of YBX1 from AsPC-1 cells transfected with SGCTR or *GSDME*-SGs and treated with Try/Chy for 36 h. Scale bar, 5 μm. **f**, The cell viability of AsPC-1 cells transfected with SGCTR, *GSDME*-SG or *GSDME*-SG/NLS-*YBX1* was determined by TB staining. **g**, AsPC-1 cells transfected with SGCTR, *YBX1*-SG, *YBX1*-SG/NLS-*YBX1*, *YBX1*-SG/NES-*YBX1* or *YBX1*-SG/NLS-GSDME (2.5 × 10^5^ cells) were injected into the pancreas of mice. Tumours were photographed (left) and weighed (right) (*n* = 6 per group). Scale bar, 1 cm. For **a**–**f**, *n* = 3 biological independent experiments. *P* values were determined one-way ANOVA Bonferroni’s test (**b**–**g**). The data represent the mean ± s.d. Source data

**Extended Data Fig. 5:**
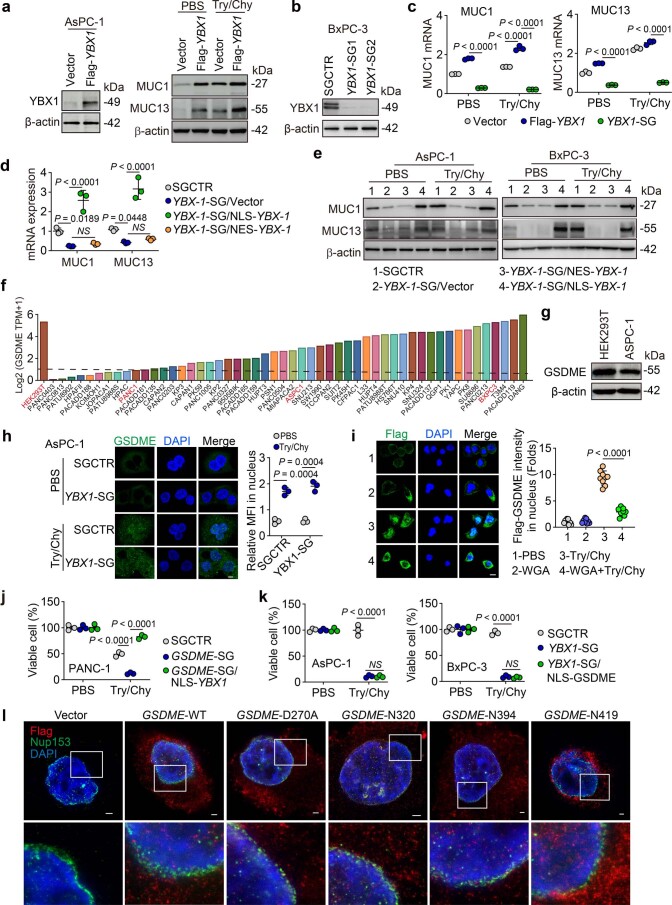
YBX1 mediated MUCs expression. **a**, The expression of YBX1, MUC1 or MUC13 from vector or Flag-YBX1-AsPC-1 cells treated with or without Trp/Chy for 48 hr was analyzed by western blot. **b**, The knockout efficiency of YBX1 from BxPC-3 cells was determined by western blot. **c-e**, The expression of MUC1 and MUC13 from SGCTR, Flag-*YBX1*, *YBX1*-SG, *YBX1*-SG/NLS-*YBX1* or *YBX1*-SG/NES-*YBX1*-AsPC-1 cells stimulated by Try/Chy for 24 hr (c, d) or 48 hr (e) was determined by real-time PCR (c, d) or western blot (e). **f**, The log2TPM + 1 expression of GSDME in HEK239T cell line and human PDAC cell lines was quantified by RNA sequencing. **g**, The level of GSDME in 293 T and AsPC-1 cells were analyzed by Western blot. **h**, Immunostaining of GSDME from SGCTR or *YBX1*-SG-AsPC-1 cells treated with Trp/Chy for 36 hr. Scale bar, 5 μm. **i**, Immunostaining of Flag from Flag*-GSDME*-AsPC-1 cells treated with Try/Chy or/and nuclear pore inhibitor WGA (100 ng/mL) for 48 hr, following SLO pretreatment, was observed under the confocal microscopy. The mean MFI of nuclear Flag was calculated by Image J. Scale bar, 10 μm. **j**, The cell viability from SGCTR, *GSDME*-SG and *GSDME*-SG/NLS-*YBX1*-PANC-1 cells treated with or without Try/Chy was determined by TB staining. **k**, SGCTR, *YBX1*-SG or *YBX1*-SG/NLS-*GSDME*-AsPC-1 or BxPC-3 cells were treated with or without Try/Chy for 72 hr. The viable cells were calculated by TB staining. **l**, Immunostaining of Flag and Nup153 from *GSDME*-SG/Vector, *GSDME*-SG/Wt-*GSDME*, *GSDME*-SG/*GSDME*-D270A, *GSDME* -SG/*GSDME*-N320, *GSDME*-SG/*GSDME*-N394 or *GSDME*-SG/*GSDME*-N419- AsPC-1 cells treated with Try/Chy for 48 hr were observed under the STEDYCON super-resolution microscope. Scale bar, 1 μm. In a-k, n = 3 biological independent experiments. ***P < 0.001, by one-way ANOVA Bonferroni’s test (**c,d**,**h-k**). The data represent mean ± SD. Source data

Next, we asked whether YBX1 in the nucleus transcriptionally regulates the expression of MUC1 and MUC13. Overexpression of YBX1 led to the upregulation of MUC1 and MUC13 in PDAC cells, whereas knockout of *YBX1* downregulated MUC1 and MUC13, even when trypsin and chymotrypsin was used (Fig. 5a and Extended Data Fig. 5a–c). In addition, in *YBX1* knockout tumour cells, replenishment of NLS-YBX1 but not NES-YBX1 upregulated MUC1 and MUC13 (Extended Data Fig. 5d,e). Moreover, only NLS-YBX1 rescued the resistance of YBX1-deficient cells to trypsin and chymotrypsin (Fig. 5b), which suggests that YBX1 regulates *MUC1* and *MUC13* in the nucleus. Chromatin immunoprecipitation (ChIP) with quantitative PCR (ChIP–qPCR) confirmed that YBX1 indeed bound to the promoters of *MUC1* and *MUC13* (Fig. 5c). HEK-293T cells, which are commonly used for exogenous gene expression^23^, also express GSDME (Extended Data Fig. 5f,g). Luciferase assays using HEK-293T cells showed that YBX1 induced MUC1 and MUC13 expression (Fig. 5d). Notably, trypsin and chymotrypsin treatment promoted the entry of YBX1 into the nucleus, which could be abolished by *GSDME* knockout (Fig. 5e). In contrast, *YBX1* knockout did not affect GSDME in the nucleus (Extended Data Fig. 5h), which, however, was blocked by the addition of the nuclear pore inhibitor wheat germ agglutinin (Extended Data Fig. 5i). Meanwhile, transfection of NLS-YBX1 into *GSDME*^−/−^ AsPC-1 and PANC-1 cells rescued the resistance to trypsin and chymotrypsin (Fig. 5f and Extended Data Fig. 5j); however, the transfection of NLS-GSDME into *YBX1*^−/−^ tumour cells did not have such an effect (Extended Data Fig. 5k). When *YBX1*^−/−^ AsPC-1 cells transfected with NLS-YBX1, NES-YBX1 or NLS-GSDME were inoculated into the pancreas of mice, the transfection of NES-YBX1 or NLS-GSDME barely rescued *YBX1*^−/−^ tumour cells to allow tumour growth. Conversely, the transfection of NLS-YBX1 favoured *YBX1*^−/−^ tumour cells and promoted tumour formation (Fig. 5g). In addition, GSDME N-terminal fragments (N320, N394 and N419) entered the nucleus, as shown by nuclear staining (Extended Data Fig. 5l). Nucleoporin 153 (NUP153) is a nuclear pore complex-associated basket protein involved in the nuclear import of proteins^24,25^. GSDME N-fragments colocalized with NUP153. However, GSDME-D270A was barely present in the nucleus, which indicates that the N-terminal part is required for the entry of GSDME into the nucleus (Extended Data Fig. 5l). Together, these results suggest that GSDME acts as a transporter to mediate the entry of YBX1 into the nucleus to promote mucin expression.

### GSDME is correlated with worse prognosis in patients with PDAC

**Fig. 6:**
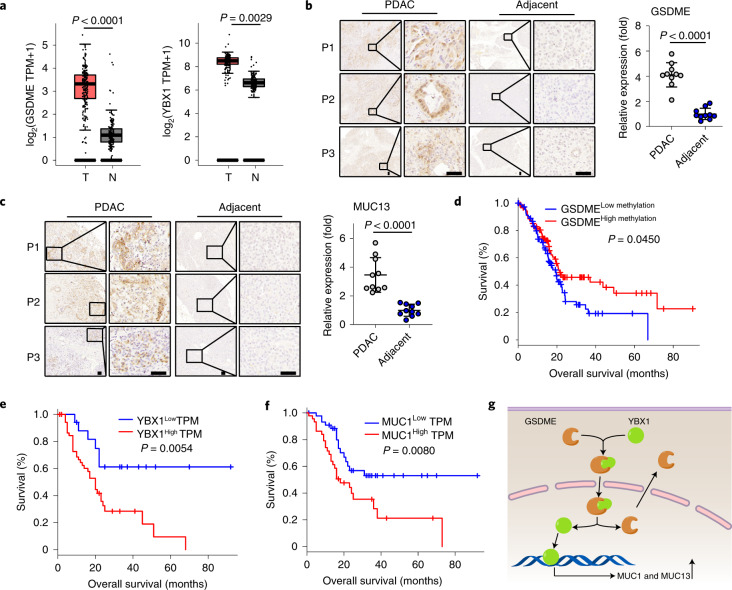
The GSDME–YBX1–mucin pathway mediates tumour progression in patients with PDAC. **a**, The expression profile of GSDME and YBX1 from the TCGA Research Network (https://cancergenome.nih.gov/). Data are presented as box plots, where the centre line shows the median, the bounds of the box show the first and third quartiles, whiskers extend to the most extreme values within 1.5-times the interquartile range, and dots denote outliers reaching past the 1.5 interquartile range. *n* = 179 for PDAC tissues and *n* = 171 for adjacent tissues. N, normal adjacent tissue; T, tumour tissues; TPM, transcripts per million. **b**,**c**, Immunohistochemical images (left) and quantification (right) of GSDME (**b**) and MUC13 (**c**) from sections of tumour tissues and adjacent tissues of patients (P1–P3) with PDAC (*n* = 10). Scale bars, 50 μm. **d**–**f**, Correlation analysis between the level of GSDME methylation (**d**), YBX1 (**e**) or MUC1 (**f**) and overall survival of patients with PDAC (*n* = 177). **g**, Schematic of the GSDME–YBX1–mucin pathway to regulate the resistance of PDAC to digestive enzymes. *P* values were determined by two-tailed Mann–Whitney test (**a**–**c**) or two-sided Pearson’s correlation test (**d**–**f**). The data represent the mean ± s.d. Source data

**Extended Data Fig. 6:**
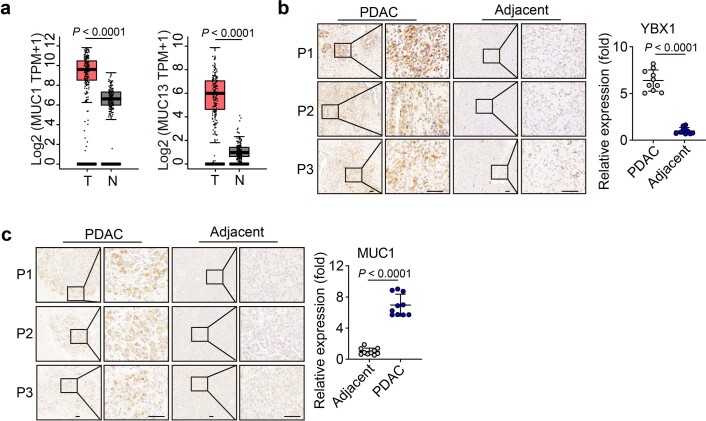
GSDME-YBX1-MUCs participated in regulating tumor growth in patients. **a**, The expression profile of MUC1 and MUC13 from the TCGA Research Network (http://cancergenome.nih.gov/). Data were presented by box plots, where the centre line shows the median, the bounds of the box show the first and third quantile, whiskers extend to the most extreme values within 1.5 interquartile range (1.5*IQR), and dots denote outliers reaching past 1.5 interquartile range. n = 179 for PDAC tissues and n = 171 for adjacent tissues. T, tumor tissues; N, adjacent normal tissues. **b**,**c**, Immunohistochemical staining of YBX1 (b) or MUC1 (c) from the pancreatic sections of PDAC patients (n = 10). Scale bar, 50 μm. **P < 0.01, ***P < 0.001, by two-tailed Mann-Whitney test (**a**-**c**). The data represent mean ± SD. Source data

Finally, we sought to validate the above-described results in patients with PDAC. Using the Gene Expression Profiling Interactive Analysis (GEPIA) database (http://gepia.cancer-pku.cn/), we analysed PDAC tumour tissues (*n* = 179) and the adjacent tissues (*n* = 171), and found that *GSDME*, *YBX1*, *MUC1* and *MUC13* mRNAs are expressed at high levels in tumour tissues compared to the adjacent tissues (Fig. 6a and Extended Data Fig. 6a). In line with these bioinformatics results, immunohistochemical staining of clinical samples from patients also revealed much higher levels of GSDME expression in PDAC tumour tissues but not in the adjacent paracancerous acinar or ductal cells, and that GSDME is present in the nucleus of the tumour cells (Fig. 6b). Consistently, YBX1, MUC1 and MUC13 were upregulated in tumour tissues of patients compared to the paracancerous tissues (Fig. 6c and Extended Data Fig. 6b,c). Moreover, using data from The Cancer Genome Atlas (TCGA) database (https://tcga-data.nci.nih.gov/), Kaplan–Meier analysis of the survival of patients with PDAC showed that the level of *GSDME* methylation positively correlated with overall survival (*P* = 0.045) (Fig. 6d). In addition, the expression of YBX1 and MUC1 was inversely correlated with overall survival of patients (*P* = 0.0054 and *P* = 0.008, respectively) (Fig. 6e,f). Together, these results suggest that the GSDME–YBX1–mucin axis in human pancreatic cancer is crucial in protecting tumour cells from enzymatic destruction.

## Discussion

Following cleavage by caspase-3, GSDME can induce tumour cell pyroptosis and enhance the antitumour immunity of T cells^15^. Thus, GSDME is considered to have a tumour-suppressing function. Consistently, GSDME is silenced in most cancer cells^11^, which can be achieved through epigenetic suppression or loss-of-function mutations in *GSDME*^15^. However, our previous studies found that GSDME is expressed at high levels in B leukaemia cells and mediates the cytokine release syndrome induced by chimeric antigen receptor T-cell therapies^26^. In this study, we further demonstrated that PDAC tumour cells express GSDME at high levels. GSDME appears to function as a transporter to mediate the entry of the transcription factor YBX1 to the nucleus, where it promotes the expression of MUC1 and MUC13. These membrane-associated mucins protect PDAC cells from cytolysis caused by digestive enzymes secreted by acinar and duct cells. Thus, GSDME can also play a tumour-promoting role.

In addition to preventing enzymatic digestion, GSDME plays an important part in mediating the pathogenesis of PDAC. A notorious pathological feature of PDAC is its strong desmoplastic stroma, which poses a formidable obstacle for treatments^27,28^. It is unclear how the desmoplastic stroma is triggered at the beginning of PDAC formation. The oncogenic *KRAS* mutation is thought to be a driving force for the PDAC desmoplasia^29–31^. Chronic inflammation is also thought to play a critical role in the stromal formation of PDAC^32^. However, what triggers and maintains pancreatic inflammation is unclear. Our present study may provide a clue, and we propose the following scenarios: (1) KRAS-transformed pancreatic epithelial cells rapidly proliferate and obstruct the normal ducts, which leads to pancreatic juice leaking to surrounding cells; (2) digestive enzymes in the pancreatic juice are activated and cytolyse normal cells; (3) transformed cells develop an increased GSDME phenotype, which facilitates their resistance to enzymatic digestion; and (4) normal cell lysis releases damage-associated molecular patterns, which trigger innate immune responses and inflammation. Future investigation is warranted to substantiate these ideas.

As the most ancient gasdermin, the conservation of GSDME in the lancelet and even earlier metazoa implies that it probably exerts a highly specific function for cell survival and propagation^11,33^. The original identification of GSDME as DFNA5 (deafness, autosomal dominant 5) was from a family with autosomal-dominant progressive hearing loss^34^. The connection of deafness to the known pore-forming function of GSDME is difficult to reconcile. However, our findings provide some answers. GSDME may have ‘moonlighting’ functions that are pyroptosis-independent and cleavage-independent but involved in transcription regulation. Our cell viability assays showed that knockout of *GSDME* abrogated the resistance of PDAC tumour cells to pancreatic digestive enzymes (Fig. 4f,g), which suggests that GSDME is required for cell survival rather than the known pore-formation role that causes cell death. Intriguingly, the D270 site, which is required for the cleavage of GSDME by activated caspase-3 to generate the N-terminal active form for membrane pore formation^11^, is also required for GSDME binding to YBX1 (Fig. 4e,f and Extended Data Fig. 4h). Based on previous studies of GSDME and our findings here, we propose that the previously known GSDME cleavage site (D270) may biologically act as a switch to guide GSDME towards either the pore-forming pathway or the YBX-1-binding pathway, which depends on the status of caspase-3 and YBX1 in the cells. In support of this moonlighting function of GSDME, it has been reported that another gasdermin (GSDMB) is localized in the nucleus of bronchial epithelial cells related to asthma-related gene expression, which suggests that gasdermins might have transcriptional roles^35^. Providing stronger data should be useful to support this notion. Together, a comprehensive understanding of the function of GSDME is worthy of further investigation.

In summary, the data in this study show that GSDME, by virtue of its upregulation in PDAC tumour cells, mediates the transcription factor YBX1 to enter the nucleus where it promotes mucin expression, which in turn enables tumour cells to escape pancreatic enzymatic digestion (Fig. 6g). These findings shed light on potential innovative strategies to target PDAC. One is to target the GSDME–YBX1–mucin pathway to deprive PDAC cells of their resistance to enzymatic digestion; another is to motivate the pore-forming activity of GSDME to trigger tumour cell pyroptosis and to activate antitumour immune responses.

## Methods

The research conducted as part of this manuscript complies with all of the relevant ethical regulations. All studies involving mice were approved by the Animal Care and Use Committee (IACUC) of the Chinese Academy of Medical Science (ACUC-A02-2020-009). The maximum tumour size allowed by the IACUC is 20 mm, and none of the experiments exceeded this limit. Human material was obtained from the Department of Surgery, Peking Union Medical College Hospital. Ethics permission was granted by the Medical Ethics Committee of Peking Union Medical College (2021105), and patients were not recruited specifically for this study. The clinical features of the patients are listed in Supplementary Table 2.

### Animals and cell lines

Female NOD-SCID mice, NSG mice and C57BL/6, 6–8 weeks old, were purchased from the Center of Medical Experimental Animals of the Chinese Academy of Medical Science. Female *Prss1*^−/−^ C57BL/6JGpt mice, 6–8 weeks old, were obtained from GemPharmatech. All the animals were maintained in the Animal Facilities of the Chinese Academy of Medical Science under specific pathogen-free conditions. All animals were placed under a 12-h light–dark cycle. The room temperature was maintained at 21 ± 1 °C with 55–70% humidity. The human pancreatic cancer cell lines PANC-1 (X100160), AsPC-1 (X100459) and BxPC-3 (X100441), the mouse pancreatic cancer cell line Pan02 (X100165), the embryonic pancreatic-tissue-derived cell line CCC-HPE-2 (X100418), HEK-293T cells (X100478) and Sf9 insect cells (X100118) were purchased from the China Center for Type Culture Collection. PANC-1, AsPC-1, BxPC-3, Pan02 and HEK-293T cells were cultured in DMEM with 10% FBS (Gibco; Thermo Fisher Scientific). CCC-HPE-2 cells were cultured in DMEM with 20% FBS (Gibco; Thermo Fisher Scientific). Cells were tested for mycoplasma detection, inter-species cross contamination and authenticated by isoenzyme and short-tandem repeat analyses in the Cell Resource Centre of Peking Union Medical College before the study. Cell lines in the experiments were used within 20 passages.

### Plasmids and reagents

pCMV-pL, pL-CRISPR.EFS.RFP and PHS-AVC in pGL4.10 were purchased from Addgene. Complementary DNA (cDNA) encoding human GSDMB and GSDME (WT, N320, N394, N419 or the uncleavable mutant D270A) were provided by F. Shao (NIBS, China). All plasmids were verified by DNA sequencing. 4′,7-Dimethoxy-5-hydroxyflavone, orlistat, PI-1840 and GO-203 were purchased from Selleck. The trypsin inhibitor α-chymotrypsin, wheat germ agglutinin, glucagon, streptolysin O and benzyl-α-GalNAc were from Sigma-Aldrich. Insulin-like growth factor 2 was from PeproTech, insulin from Gibco (Thermo Fisher), and trypsin solution from Solarbio.

### Preparation of pancreatic lysate

Fresh pancreatic tissue was thoroughly washed and homogenized in 1 ml cold sterile PBS. The homogenization solution was centrifuged at 15,000*g* for 10 min at 4 °C, followed by passing through a 0.22-μm filter. The supernatant was collected as the pancreatic lysate.

### Trypan blue staining

Cells treated with trypsin and chymotrypsin or with lysate were digested into a single-cell suspension and mixed with 0.4% trypan blue dye. The viable cells and dead cells were counted within 3 min, as the dead cells can be dyed blue. The percentage of viable cells is calculated by the ratio of the number of viable cells to total cells.

### Cell fractionation

The cytoplasmic and nuclear proteins were isolated using a Cell Fractionation kit (BioVision) according to the manufacture’s instruction. Equal cell equivalents were analysed by western blotting.

### Cell viability detection

Cell viability was measured using a CellTiter-Glo Luminescent Cell Viability Assay kit (Promega), which is based on the luciferase reaction to measure the amount of ATP from viable cells. The amount of ATP in the cells correlates with cell viability.

### Luciferase assays

HEK-293T cells were transfected with 100 ng Renilla luciferase plasmid (pRL-SV40), 1 μg firefly luciferase plasmid (pGL4.10-MUC1 or pGL4.10-MUC13) and 1 μg of pCMVh-YBX1 plasmid for 12 h. The cells were then treated with trypsin (50 U ml^–1^) and chymotrypsin (40 U ml^–1^) for 24 h. Cell lysates were analysed using a Dual-Luciferase Reporter assay (Promega) on a GloMax Multi Plus (Promega). Firefly luciferase activity was normalized to Renilla luciferase.

### PCR with reverse transcription

TRIzol (Invitrogen, 15596018) was used to extract total RNA from cells, which was then transcribed to cDNA using a high-capacity cDNA reverse transcription kit (Applied Biosystems, 4368813). Real-time PCR was performed using ABI stepone plus (Applied Biosystems). The following primer sequences were used: *MUC1*, 5′-TGCCGCCGAAAGAACTACG-3′ (sense) and 5′-TGGGGTACT CGCTCATAGGAT-3′ (antisense); *MUC3A*, 5′-CTCCCAGACCCTGTGTTTTAAG-3′ (sense) and 5′-ACCAAGGGGAAGTAGAACTCTT-3′ (antisense); *MUC4*, 5′-CGTTCTGGGACGATGCTGAC-3′ (sense) and 5′-GATGGCTTGGTAGGTGTT GCT-3′ (antisense); *MUC12*, 5′-CCAGTTCAAGCGACCCTTTTA-3′ (sense) and 5′-CGCTGTGGGATACTGTTGA TT-3′ (antisense); *MUC13*, 5′-ATGCGTGCTGATGACAAGTTT-3′ (sense) and 5′-ACACCGAAGGGTCAAATCATAGT-3′ (antisense); *MUC15*, 5′-TATTCACTTCTATCGGGGAGCC-3′ (sense) and 5′-GGGAATGACTCGCCTTGAGAT-3′ (antisense); *MUC16*, 5′-CCAGTCCTACATCTTCGGTTGT-3′ (sense) and 5′-AGGGTAGTTCCTAGAGGGAGTT-3′ (antisense); *TET1*, 5′-CATCAGTCAAGACTTTAAGCCCT-3′ (sense) and 5′-CGGGTGGTTTAGGTTCTGTTT-3′ (antisense); *TET2*, 5′-GGCTACAAAGCTCCAGAATGG-3′ (sense) and 5′-AAGAGTGCCACTTGGTGTCTC-3′ (antisense); *TET3*, 5′-TCCAGCAACTCCTAG AACTGAG-3′ (sense) and 5′-AGGCCGCTTGAATACTGACTG-3′ (antisense); *YBX1*, 5′-CCCCAGGAAGTACCTTCGC-3′ (sense) and 5′-AGCGTCTATAATGGTTACGGTCT-3′ (antisense); *GSDME*, 5′-TGCCTACGGTGTCATTGAGTT-3′ (sense) and 5′-TCTGGCATGTCTATGAATGCAAA-3′ (antisense); and *GAPDH*, 5′-TGGCCTTCCGTGTTCCTAC-3′ (sense) and 5′-GAGTTGCTGTTGAAGTCGCA-3′ (antisense). The results were analysed using QuantStudio Design and Analysis software 1.5. Values are the mean ± s.d. from three independent experiments that were performed in duplicate.

### Western blot assays

Cells were collected, lysed in M2 lysis buffer and sonicated. The protein concentrations were determined by a BCA kit (Applygen Technologies). The protein was run on a SDS–PAGE gel and transferred to a nitrocellulose membrane. Nitrocellulose membranes were blocked in 5% BSA and probed with the following antibodies overnight: anti-β-actin (Cell Signaling Technology, 3700S; clone 8H10D10); anti-YBX1 (Cell Signaling Technology, 4202S; clone D299); MUC1 (Abcam, ab45167; clone EP1024Y); GSDME (Abcam, ab215191; clone EPR19859); MUC13 (Abcam, ab65109); TET2 (Abcam, ab94580); anti-β3-tubulin (Cell Signaling Technology, 631836); anti-histone 3 (Cell Signaling Technology, 4499S; clone D1H2); and anti-Flag (Sigma, F1804; clone M2). Secondary antibodies conjugated to horseradish peroxidase (1:3,000) were followed by enhanced chemiluminescence (Thermo Fisher). Results were confirmed by at least three independent experiments.

### ChIP–qPCR assays

ChIP–qPCR was performed using a MAGnity ChIP system (Invitrogen) according to the manufacturer’s protocol. In brief, cells were crosslinked and chromatin was extracted and sheared. Samples were immunoprecipitated with anti-5hMC antibody (Cell Signaling Technology, 51660S; clone HMC31) or anti-YBX1 antibody (Cell Signaling Technology, 9744; clone D2A11). The primer sequences used for ChIP–qPCR are as follows: *GSDME*, 5′-GACCCCTACTGCACTTCTGCAC-3′ (sense) and 5′-TGGGAAGAGACAGAGCCAAGAT-3′ (antisense); *MUC1*, 5′- CGGCCTGGGATA GCTTCCTC-3′ (sense) and 5′-TGCACTCCAGCCTGGGCG-3′ (antisense); and *MUC13*, 5′- GGAACTAGAGAGAGGGTGAGAAAGGGA-3′ (sense) and 5′-CTCTCTGATGGTCATGTCTAGCAAC-3′ (antisense). The results were from three independent experiments followed by normalization to input signals and shown as the mean ± s.d..

### Immunofluorescence

Cells were fixed in 4% paraformaldehyde and permeabilized with 0.2% Triton X-100. Fixed cells were blocked in 5% BSA and incubated with anti-GSDME (GeneTex, GTX81693, 1:100), anti-YBX1 (GeneTex, GTX81909, 1:100), anti-TET1 (GeneTex, GTX124207; clone N3C1, 1:100), anti-TET2 (Abcam, ab94580; 1:100) and anti-TET3 (GeneTex, GTX00657; 1:100), anti-Nup153 (Abcam, ab84872; 1:100) and anti-Flag (Sigma, F1804; clone M2, 1:100) at 4 °C overnight. Cells were then washed and incubated with secondary antibodies for 1 h at room temperature. The slides were counterstained with 4,6-diamidino-2-phenylindole (DAPI) and mounted for confocal analysis. The intensity of immunofluorescence was analysed using ImageJ 9.0 software.

### Generation of CRISPR–Cas9 knockout cell lines

For construction of stable knockdown of MUC1, MUC13, GSDME, YBX1, or TET2- BxPC-3, PANC-1 or AsPC-1 cells, the following sgRNAs targeting *MUC1*, *MUC13*, *GSDME*, *YBX1* or *TET2* were used: control sgRNA (SGCTR), CACCGGGGCGAGGAGCTGTTCACCG (sense) and AAACCGGTGAACAGCTCCTCGCCCC (antisense); *MUC1*-SGRNA1 (human), CACCGCATGCAAGCTCTACCCCAGG (sense) and AAACCCTGGGGTAGAGCTTGCATGC (antisense); *MUC1*-SGRNA2 (human), CACCGAGGTGGAGAAAAGGAGACTT (sense) and AAACAAGTCTCCTTTTC TCCACCTC (antisense); *MUC13*-SGRNA1 (human), CACCGACCACAGAAACT GCGACTAG (sense) and AAACCTAGTCGCAGTTTCTGTGGTC (antisense); *MUC13*-SGRNA2 (human), CACCGCAGAAACTGCGACTAGTGGT (sense) and AAACACCACTAGTCGCAGTTTCTGC (antisense); *GSMDE*-SGRNA1 (human), CACCGGTCGGACTTTGTGAAATACG (sense) and AAACCGTATTTCACAAAGT CCGACC (antisense); *GSMDE*-SGRNA2 (human), CACCGGAACCCTGGAGAC TGCACTG (sense) and AAACCAGTGCAGTCTCCAGGGTTCC (antisense); *Gsmde*-SGRNA1 (mice), CACCGAAAAGAAGAGATACTGGTGC (sense) and AA ACGCACCAGTATCTCTTCTTTTC (antisense); *Gsmde*-SGRNA2 (mice), CACCGTGTGAGTACATCTTCCAGGG (sense) and AAACCCCTGGAAGATGTACTCACAC (antisense); *YBX1*-SGRNA1 (human), CACCGTCAGCGCCGCCGACACCAAG (sense) and AAACCTTGGTGTCGGCGGCGCTGAC (antisense); *YBX1*-SGRNA2 (human), CACCGCCGACACCAAGCCCGGCACT (sense) and AAACAGTGCCGG GCTTGGTGTCGGC (antisense); *TET2*-SGRNA1 (human), CACCGACCATGTTGAGGGCAACAGA (sense) and AAACTCTGTTGCCCTCAACATGGTC (antisense); and *TET2*-SGRNA2 (human), CACCGTGTTGAGGGCAACAGACTAA (sense) and AAACTTAGTCTGTTGCCCTCAACAC (antisense). These sgRNAs were cloned into the pL-CRISPR.EFS.RFP vector plasmid (Addgene, 57819) and transfected into HEK-293T cells together with the packing plasmids psPAX2 and pMD2.G. After 48 h, the lentivirus was collected and concentrated to infect BxPC-3, PANC-1 or AsPC-1 cells together with polybrene at a final concentration of 8 μg ml^–1^. After 48 h, RFP-positive cells were sorted by flow cytometry using a BD Biosciences FACSAria III. Candidate knockout cells were verified by western blotting.

### Generation of humanized mice

Immune-deficient NSG mice were injected with 1 × 10^5^ CD34^+^ human haematopoietic stem cells 12 h after sublethal irradiation with 3.5 Gy. The peripheral lymphocytes were analysed by FACS after 8–10 weeks using an APC anti-human CD45 antibody (BioLegend, 304012; clone HI30) to check for the reconstitution of the human immune system. Flowjo v.10 was used to analyse the flow cytometry data.

### Proximity ligation assay

Proximity ligation assays (PLAs) were performed on Flag–GSDME-overexpressing AsPC-1 cells grown on glass coverslips using the Duolink in situ PLA kit according to the manufacturer’s instructions (Sigma). In brief, cells were fixed with 4% paraformaldehyde for 15 min and permeabilized with 0.1% Triton X-100. After blocking, the cells were incubated overnight at 4 °C with anti-Flag (Sigma, F1804; clone M2) and anti-YBX1 (Cell Signaling Technology, 4202S; clone D299) antibodies. PLA Plus and Minus probes for mouse and rabbit antibodies were added and incubated for 1 h at 37 °C. The proximity ligation reaction was performed using oligonucleotides and ligase for 30 min at 37 °C. Samples were counterstained with DAPI (Thermo) and mounted for confocal analysis, in which cells were scanned and digitalized utilizing a Nikon A1 confocal microscope. Red fluorescent dots were analysed and quantified using ImageJ v.1.52 software.

### RNA immunoprecipitation

RNA immunoprecipitation (RIP) experiments were performed using a Magna RIP kit (Millipore). In brief, Flag–GFP-overexpressing AsPC-1 or Flag–GSDME-overexpressing AsPC-1 cells were lysed and immunoprecipitated using anti-Flag antibody, which was pre-bound to protein A agarose beads, with normal mouse IgG as a negative control. The precipitated mRNA was converted to cDNA and analysed by real-time PCR.

### Stably overexpressing MUC1, MUC13, GSDME and YBX1

The cDNAs for *MUC1*, *MUC13*, *GSDME* and *YBX1* were purchased from Sino-Biological (MUC1, HG12123-UT; MUC13, HG21326-UT; GSDME, HG19167-UT; YBX1, HG17046-UT). These cDNAs with or without the NLS or the NES were inserted into the lentiviral vector plasmid pLV-EF1α-IRES-Puro (Addgene, 85132) with a carboxy-terminal 3×Flag tag for transient expression in 293T cells to obtain the lentivirus containing the target gene. The lentiviruses containing NLS, WT or NES *GSDME* or *YBX1*, *MUC1* or *MUC13* were transduced with *GSDME*-SGs or *YBX1*-SGs AsPC-1, PANC-1 or BxPC-3 cells. These infected cells were then cultured with 2 μg ml^–1^ puromycin to select cell clones with high expression of target genes. The efficiency of overexpression of target genes was confirmed by western blotting.

### Recombinant GSDME and YBX1

GSDME with a C-terminal 6×His tag was subcloned into pFastBac1 (presented by F. Shao) to construct the recombinant plasmid of pFastBac1-GSDME. Then, DH10Bac *Escherichia coli* cells (provided by F. Shao) were transformed with this plasmid to obtain a recombinant bacmid. Sf9 insect cells were transfected with the recombinant bacmid using Cellfectin II (Gibco) according to the manufacturer’s instructions. Then, his-GSDME was purified from the cell lysates of baculovirus-infected Sf9 cells. The purity of the purified GSDME was assessed by electrophoresis on a 10% polyacrylamide gel, and a 55-kDa band was visualized by silver staining and immunoblotting. YBX1 with a C-terminal 6×His tag was treated as same as GSDME, and a 49-kDa band was visualized by silver staining and immunoblotting.

### Histological and immunohistochemical staining

Pancreatic tumour tissues from patients were embedded in paraffin and sectioned for immunohistochemical staining. Immunohistochemistry was performed using a DAB horseradish peroxidase colour development kit (ZSGB-BIO) according to the manufacturer’s instructions. In brief, the sections of paraffin-embedded tissues were incubated with anti-MUC1 (Abcam, ab45167; 1:1,000), anti-MUC13 (Abcam, ab124654; 1:200), anti-GSDME (GeneTex, GTX81693, 1:100) or anti-YBX1 (GeneTex, GTX81909, 1:100) at 4 °C overnight. Then, slides were sequentially incubated with two HRP-conjugated secondary antibodies for 1 h at room temperature. The stained sections were scanned and digitalized utilizing a TissueFaxs Plus System coupled onto a Zeiss Axio Imager Z2 microscope. The intensity of positive staining was analysed using ImageJ 9.0 software.

### Bioinformatics analysis

The GEPIA dataset (http://gepia.cancer-pku.cn/) was used to analyse TCGA tumours versus TCGA normal + the genotype-tissue expression (GTEx) normal datasets, and the box plots for the expression of YBX1, GSDME, MUC1 and MUC13 between pancreatic adenocarcinoma (*n* = 179) and the adjacent tissues (*n* = 171) were obtained. The log_2_ fold-change cut-off was set as 1 and the *P* value cut-off was 0.01. Genes with higher |log_2_ fold-change| values and lower *q* values than the preset thresholds were considered differentially expressed genes^36^. For survival analysis, the Kaplan–Meier plotter database was used according to the GSDME, YBX1 and MUC1 expression in the GEPIA database. In addition, we downloaded the methylation data of GSDME from the TCGA database and used the R package survminer to analyse the relationship between GSDME methylation level and prognosis.

### Animal experiments and treatment protocol

Female NOD-SCID mice (6–8 weeks) were orthotopically injected with cells transfected with SGCTR, *GSDME*-SG or Flag-*GSDME*-AsPC-1 (5 × 10^5^ cells per mouse); cells transfected with SGCTR, *GSDME*-SGs, *GSDME*-SG/Flag-*GSDME*, *MUC1*-SG, *MUC13*-SG, *MUC1/MUC13*-SGs, *YBX1*-SGs, *YBX1*-SG/Flag-*MUC1/MUC13*-1/2, *YBX1*-SG/NLS-*YBX1*, *YBX1*-SG/NES-*YBX1* or *YBX1*-SG/NLS-*GSDME*- AsPC-1 (2.5 × 10^5^ cells per mouse); or cells transfected with SGCTR, *GSDME*-SGs or *GSDME*-SG/Flag-*GSDME* BxPC-3 (2.5 × 10^5^ cells per mouse). Mice were randomly assigned into different groups. Some mice were treated with GO-203 (14 mg per kg) after 3 days of inoculation. The control group was treated with the same volume of PBS. Mice were killed at day 40 (for those injected with AsPC-1 or BxPC-3 cells) or day 60 (for those injected with PANC-1 cells). Pancreatic tumours were weighed and survival time of the mice was recorded. In some experiments, female NOD-SCID were subcutaneously injected with WT AsPC-1 cells, or PANC-1 cells or BxPC-3 cells transfected with SGCTR or *GSDME*-SGs (2 × 10^6^ cells). The tumour growth was calculated and the mice survival was recorded. The tumour volume was calculated using the formula tumour length × tumour width^2^/2.

### Statistics and reproducibility

All experiments were performed with at least three biological repeats except indicated in the figure legends, and no statistical method was used to predetermine sample sizes. No data were excluded from analyses. The mice were randomly assigned to different groups. Analyses were conducted using Graphpad Prism 8.0 software. Results are expressed as the mean ± s.d. as indicated, and analysed using Student’s *t*-test followed by two-tailed paired *t*-test or Mann–Whitney test or one-way analysis of variance (ANOVA) followed by Bonferroni as indicated. *P* < 0.05 was considered statistically significant. To analyse the correlation between the level of GSDME methylation, YBX1 and MUC1 and the overall survival of patients, two-sided Pearson’s correlation test was applied. The survival rates were evaluated using log-rank test.

### Reporting Summary

Further information on research design is available in the Nature Research Reporting Summary linked to this article.

## Online content

Any methods, additional references, Nature Research reporting summaries, source data, extended data, supplementary information, acknowledgements, peer review information; details of author contributions and competing interests; and statements of data and code availability are available at 10.1038/s41556-022-00857-4.

## Supplementary information

Reporting Summary

Supplementary Tables Supplementary Table 1: *GSDME*-SG/Flag-*GSDME*-AsPC-1 cells were treated with Try/Chy for 48 h. Cell lysates were immunoprecipitated with anti-Flag or anti-IgG antibody for mass spectrometry. Supplementary Table 2: Clinical information of patients with PDAC.

## Data Availability

All data needed to evaluate the conclusions are present in the paper or the Supplementary Information. The mass spectrometry data reported in this paper have been deposited in the ProteomeXchange with the primary accession code PXD030879 (http://proteomecentral.proteomexchange.org/cgi/GetDataset). The human pancreatic adenocarcinoma data were derived from the TCGA Research Network (https://cancergenome.nih.gov/). All other data supporting the findings of this study are available from the corresponding author on reasonable request. Source data are provided with this paper.

## Source data

Source Data Fig. 1 Statistical source data.
Source Data Fig. 1 Unprocessed western blots.
Source Data Fig. 2 Statistical source data.
Source Data Fig. 3 Statistical source data.
Source Data Fig. 3 Unprocessed western blots.
Source Data Fig. 4 Statistical source data.
Source Data Fig. 4 Unprocessed western blots.
Source Data Fig. 5 Statistical source data.
Source Data Fig. 5 Unprocessed western blots.
Source Data Fig. 6 Statistical source data.
Source Data Extended Data Fig. 1 Statistical source data.
Source Data Extended Data Fig. 1 Unprocessed western blots.
Source Data Extended Data Fig. 2 Statistical source data.
Source Data Extended Data Fig. 2 Unprocessed western blots.
Source Data Extended Data Fig. 3 Statistical source data.
Source Data Extended Data Fig. 3 Unprocessed western blots and gels.
Source Data Extended Data Fig. 4 Statistical source data.
Source Data Extended Data Fig. 4 Unprocessed western blots.
Source Data Extended Data Fig. 5 Statistical source data.
Source Data Extended Data Fig. 5 Unprocessed western blots.
Source Data Extended Data Fig. 6 Statistical source data.
