# Supplementary material for: Gasdermin E mediates resistance of pancreatic adenocarcinoma to enzymatic digestion through a YBX1–mucin pathway
Source: Nat Cell Biol. 2022 Mar 15;24(3):364–72. doi: 10.1038/s41556-022-00857-4 (PMC8924000; doi:10.1038/s41556-022-00857-4)
Supplement: Supplementary file 1 — Reporting Summary [file 41556_2022_857_MOESM1_ESM.pdf]

nature portfolio

Corresponding author(s): Bo Huang

Last updated by author(s): Jan 11, 2022

Reporting Summary

Nature Portfolio wishes to improve the reproducibility of the work that we publish. This form provides structure for consistency and transparency in reporting. For further information on Nature Portfolio policies, see our Editorial Policies and the Editorial Policy Checklist.

Statistics

For all statistical analyses, confirm that the following items are present in the figure legend, table legend, main text, or Methods section.

n/a	Confirmed	
☐	☒	The exact sample size (*n*) for each experimental group/condition, given as a discrete number and unit of measurement
☐	☒	A statement on whether measurements were taken from distinct samples or whether the same sample was measured repeatedly
☐	☒	The statistical test(s) used AND whether they are one- or two-sided<br>*Only common tests should be described solely by name; describe more complex techniques in the Methods section.*
☒	☐	A description of all covariates tested
☒	☐	A description of any assumptions or corrections, such as tests of normality and adjustment for multiple comparisons
☐	☒	A full description of the statistical parameters including central tendency (e.g. means) or other basic estimates (e.g. regression coefficient) AND variation (e.g. standard deviation) or associated estimates of uncertainty (e.g. confidence intervals)
☐	☒	For null hypothesis testing, the test statistic (e.g. *F*, *t*, *r*) with confidence intervals, effect sizes, degrees of freedom and *P* value noted<br>*Give P values as exact values whenever suitable.*
☒	☐	For Bayesian analysis, information on the choice of priors and Markov chain Monte Carlo settings
☒	☐	For hierarchical and complex designs, identification of the appropriate level for tests and full reporting of outcomes
☐	☒	Estimates of effect sizes (e.g. Cohen's *d*, Pearson's *r*), indicating how they were calculated

Our web collection on statistics for biologists contains articles on many of the points above.

Software and code

Policy information about availability of computer code

Data collection	R package- survminer; BD Biosciences FACSAria III.
Data analysis	GraphPad Prism 8.0.0 was used for analysis of in vivo and in vitro phenotypic assays and for producing graphs. ImageJ v. 1.52 was used for the visualization and presentation of western blots, and analysis of immunohistochemical staining. QuantStudio Design & Analysis Software 1.5 was used to analyse the qPCR results. Flowjo v.10 was used to analyse the flow cytometry data.

For manuscripts utilizing custom algorithms or software that are central to the research but not yet described in published literature, software must be made available to editors and reviewers. We strongly encourage code deposition in a community repository (e.g. GitHub). See the Nature Portfolio guidelines for submitting code & software for further information.

Data

Policy information about availability of data

All manuscripts must include a data availability statement. This statement should provide the following information, where applicable:

- Accession codes, unique identifiers, or web links for publicly available datasets
- A description of any restrictions on data availability
- For clinical datasets or third party data, please ensure that the statement adheres to our policy

All data needed to evaluate the conclusions are present in the paper or the Supplementary Materials. The mass spectrometry data reported in this paper have been deposited in the ProteomeXchange. The human pancreatic adenocarcinoma data were derived from the TCGA Research Network(http://cancergenome.nih.gov/). Source data are provided with this paper. All other data supporting the findings of this study are available from the corresponding author on reasonable request.

Field-specific reporting

Please select the one below that is the best fit for your research. If you are not sure, read the appropriate sections before making your selection.

☒ Life sciences ☐ Behavioural & social sciences ☐ Ecological, evolutionary & environmental sciences

For a reference copy of the document with all sections, see nature.com/documents/nr-reporting-summary-flat.pdf

Life sciences study design

All studies must disclose on these points even when the disclosure is negative.

Sample size	Sample sizes were determined on the basis of estimates from preliminary experiments.
Data exclusions	No data were excluded from the analysis.
Replication	At least three biologically independent experiments were performed in each case, unless otherwise stated in the respective figure legend. Replicates were reproducible.
Randomization	For the animal study, mice were randomly divided into different groups to investigated tumor growth. The tests were also randomly selected from all samples. The pictures were representatively shown. Samples receiving in vitro treatments were completely randomized.
Blinding	Patients- and mice- tissue staining were performed blindly. For RNA and histologic quantification, performer was blinded to specimen/genotype during data collection and analysis.

Reporting for specific materials, systems and methods

We require information from authors about some types of materials, experimental systems and methods used in many studies. Here, indicate whether each material, system or method listed is relevant to your study. If you are not sure if a list item applies to your research, read the appropriate section before selecting a response.

Materials & experimental systems

n/a	Involved in the study	
☐	☒	Antibodies
☐	☒	Eukaryotic cell lines
☒	☐	Palaeontology and archaeology
☐	☒	Animals and other organisms
☐	☒	Human research participants
☒	☐	Clinical data
☒	☐	Dual use research of concern

Methods

n/a	Involved in the study	
☒	☐	ChIP-seq
☐	☒	Flow cytometry
☒	☐	MRI-based neuroimaging

Antibodies

Antibodies used

The following antibodies were used in Western blot:
anti-β-actin (Cell signaling technology, Cat.: 3700S; Clone: 8H10D10; Lot: 18; 1:1,000),
anti-YBX1 (Cell Signaling Technology, Cat.: 4202S; 1:1,000),
anti-MUC1 (Abcam, Cat.: ab45167; Lot: GR3191282-6; 1:1,000),
anti-GSDME (Abcam, Cat.: ab215191; Lot: GR3187594-14; 1:1,000),
anti-MUC13 (Abcam, Cat.: ab65109; Lot: GR36953-27; 1:1,000),
anti-TET2 (Abcam, Cat.: ab94580; Lot: GR3243631-1; 1:1,000),
anti-β-Tubulin (Cell signaling technology, Cat.: 2128S; Clone: 9F3; 1:1,000),
anti-Histone3 (Cell signaling technology, Cat.: 4499S; 1:1,000);
anti-Flag (Sigma, Cat.: F1804; Lot: SLCF4933; 1:1,000);
The following antibodies were used in immunofluorescence:
anti-GSDME (GeneTex, Cat: GTX81693, 1:100),
anti-YBX1 (GeneTex, Cat: GTX81909; Lot: 821902110; 1:100),
anti-TET1 (GeneTex, Cat: GTX124207; 1:100),
anti-TET2 (Abcam, Cat: ab94580; Lot: GR3243631-1; 1:100)
anti-TET3 (GeneTex, Cat: GTX00657; 1:100),
anti-Nup153 (Abcam, Cat: ab84872; 1:100)
anti-Flag (Sigma, Cat: F1804; clone: M2, 1:100) ;
Immunohistochemistry:
anti-mucin 1 (Abcam, Cat: ab45167; 1:1,000),
anti-mucin 13 (Abcam, Cat: ab124654; Lot: GR119772-58; 1:200),
anti-GSDME (GeneTex, Cat: GTX81693; 1:100)

	anti-YBX1 (GeneTex, Cat.: GTX81909; 1:100) ;<br>Flow cytometry: APC anti-human CD45 antibody (Biolegend, Cat.: 304012; clone: HI30);<br>ChIP: anti-5hMC (Active motif, Cat: 40900; 1:50)
Validation	All antibodies have been validated by the manufacturer. Antibodies used for Western blot were validated by their manufacture companies. These antibodies are routinely used in our laboratory.

Eukaryotic cell lines

Policy information about cell lines

Cell line source(s)	Human pancreatic cancer cell lines PANC-1 (Cat.: X100160), AsPC-1 (Cat.: X100459) and BxPC-3 (Cat.: X100441), mice pancreatic cancer cell line Pan02 (Cat.: X100165), embryonic pancreatic tissue-derived cell line CCC-HPE-2 (Cat.: X100418), HEK-293T (Cat.: X100478), and Sf9 insect cells (Cat.: X100118) were purchased from China Center for Type Culture Collection (Beijing, China).
Authentication	Cells were tested for mycoplasma detection, inter-species cross contamination and authenticated by isoenzyme and short-tandem repeat (STR) analyses in the Cell Resource Centre of Peking Union Medical College before the study.
Mycoplasma contamination	Our cell lines are routinely tested for mycoplasma. None of the cell lines used in this study have tested positive for mycoplasma.
Commonly misidentified lines<br>(See ICLAC register)	No commonly misidentified cell lines were used.

Animals and other organisms

Policy information about studies involving animals; ARRIVE guidelines recommended for reporting animal research

Laboratory animals	Female NOD-SCID mice, NSG mice and C57BL/6, 6-8 weeks old, were purchased from the Center of Medical Experimental Animals of the Chinese Academy of Medical Science (Beijing, China). Female Prss1-/- C57BL/6JGpt mice, 6-8 weeks old were obtained from GemPharmatech Co., Ltd, China. These animals were maintained in the Animal Facilities of Chinese Academy of Medical Science under pathogen-free conditions. All animals were placed under a 12-h light–dark cycle. Room temperature was maintained at 21 ± 1 °C with 55–70% humidity.
Wild animals	No Wild animals were used in this study.
Field-collected samples	No Field-collected samples were used in this study.
Ethics oversight	These animals were maintained in the Animal Facilities of Chinese Academy of Medical Science under pathogen-free conditions. All studies involving mice were approved by the Animal Care and Use Committee of Chinese Academy of Medical Science (ACUC-A02-2020-009).

Note that full information on the approval of the study protocol must also be provided in the manuscript.

Human research participants

Policy information about studies involving human research participants

Population characteristics	Human histopathological sections of PDACs (well or moderately differentiated) were obtained from the Department of Surgery, Peking Union Medical College Hospital, China. Across the entire manuscript, a total of 10 PDAC patients with age range of 48-74 and sex distribution of 4 females/6 males were used. Detailed information is available in supplementary table 2.
Recruitment	Pancreatic ductal adenocarcinoma tissues and adjacent normal tissues were used. We did not recruit donors specifically for this study. Participants did not receive compensation from the authors.
Ethics oversight	Ethical permission was granted by the Medical Ethics Committee of Peking Union Medical College.

Note that full information on the approval of the study protocol must also be provided in the manuscript.

Flow Cytometry

Plots

Confirm that:

☒ The axis labels state the marker and fluorochrome used (e.g. CD4-FITC).

☒ The axis scales are clearly visible. Include numbers along axes only for bottom left plot of group (a 'group' is an analysis of identical markers).

☒ All plots are contour plots with outliers or pseudocolor plots.

☒ A numerical value for number of cells or percentage (with statistics) is provided.

Methodology

Sample preparation	The peripheral lymphocytes were isolated by using the lymphocyte separation kit (Solarbio) from humanized mice peripheral blood.
Instrument	Accuri C6 (BD Biosciences).
Software	FlowJo software.
Cell population abundance	The proportion of human CD45 positive cells was analyzed.
Gating strategy	All gates were set based on FMO (full minus one) stains and isotype control antibodies after appropriate compensation using single-stained compensation controls. Cells were selected using FSC/SSC scatter profile to remove debris and doublets were excluded using FSC-A/FSC-H to select for single cells. The strategy is included in Extended Data Fig. 1m.

☒ Tick this box to confirm that a figure exemplifying the gating strategy is provided in the Supplementary Information.
