# Supplementary figures and images for: Gasdermin E mediates resistance of pancreatic adenocarcinoma to enzymatic digestion through a YBX1–mucin pathway
Source: Nat Cell Biol. 2022 Mar 15;24(3):364–72. doi: 10.1038/s41556-022-00857-4 (PMC8924000; doi:10.1038/s41556-022-00857-4)

Figure 1a

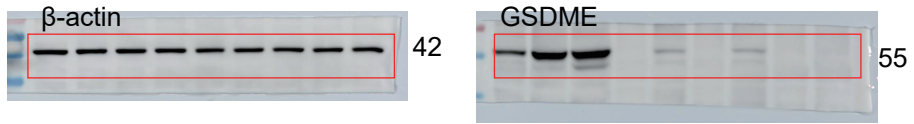

Supplement: Source Data Fig. 1 — Unprocessed western blots. [file 41556_2022_857_MOESM4_ESM.pdf]

Figure3.a

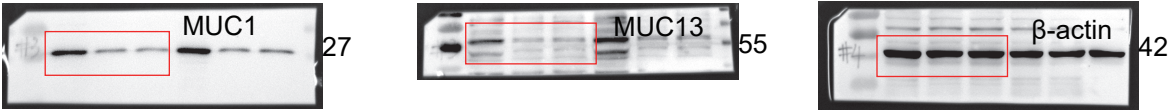

Figure3.d

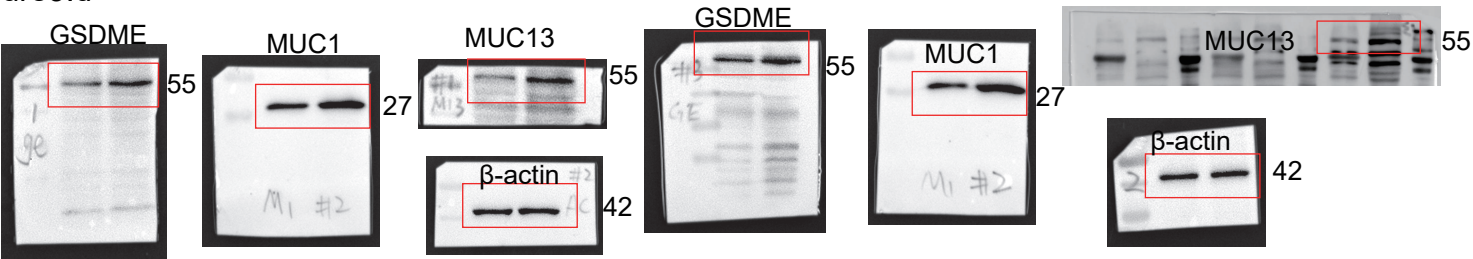

Figure3.e

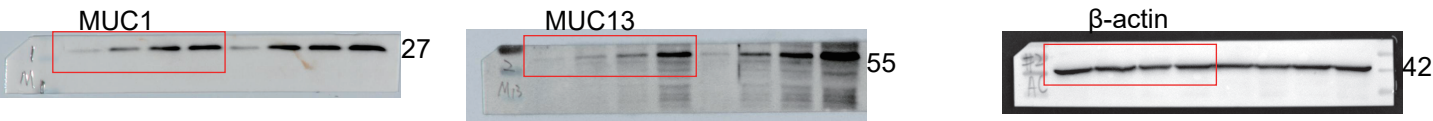

Supplement: Source Data Fig. 3 — Unprocessed western blots. [file 41556_2022_857_MOESM7_ESM.pdf]

Figure 4b

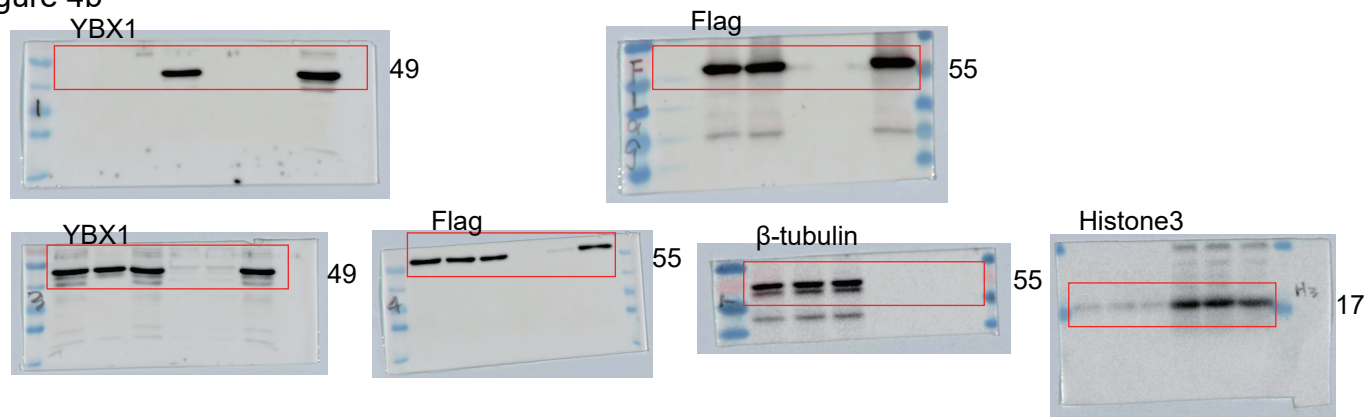

Supplement: Source Data Fig. 4 — Unprocessed western blots. [file 41556_2022_857_MOESM9_ESM.pdf]

Figure 5a

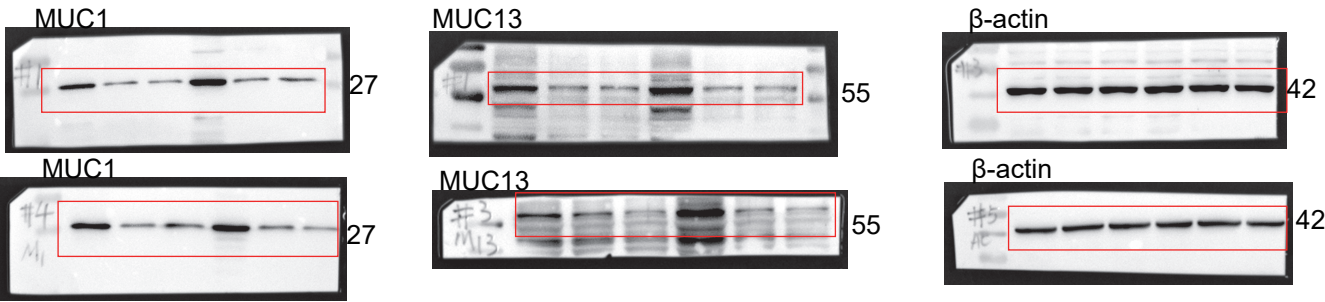

Supplement: Source Data Fig. 5 — Unprocessed western blots. [file 41556_2022_857_MOESM11_ESM.pdf]

Figure S1c

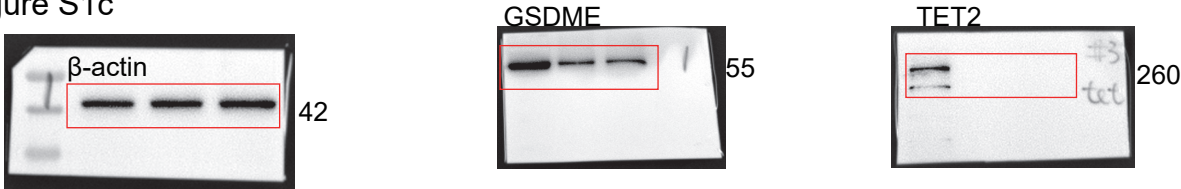

Figure S1e

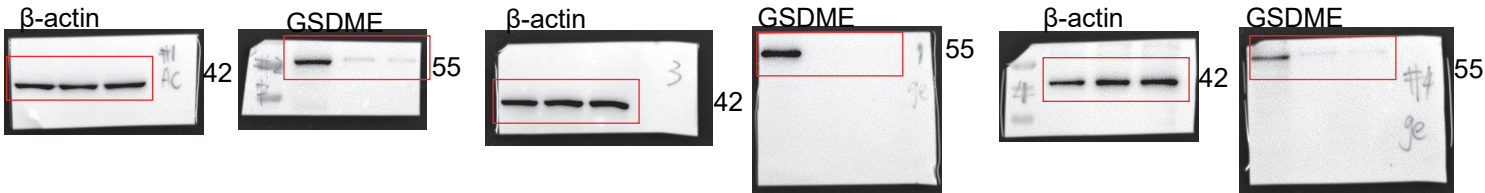

Figure S1f

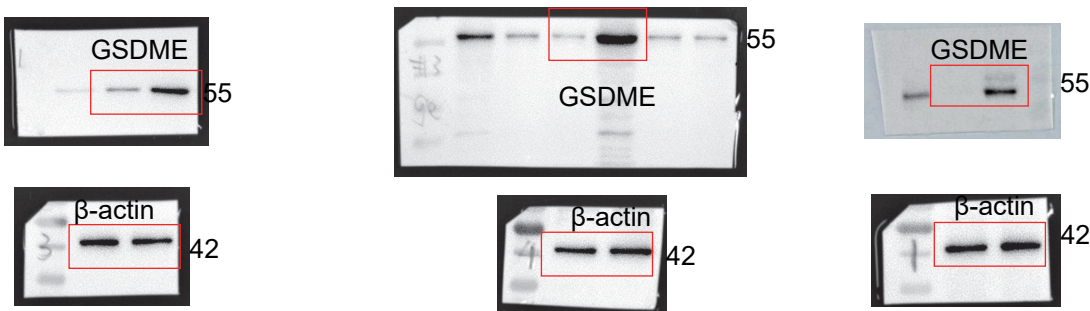

Figure S1k

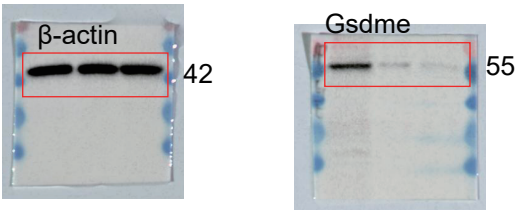

Supplement: Source Data Extended Data Fig. 1 — Unprocessed western blots. [file 41556_2022_857_MOESM14_ESM.pdf]

Figure S2h

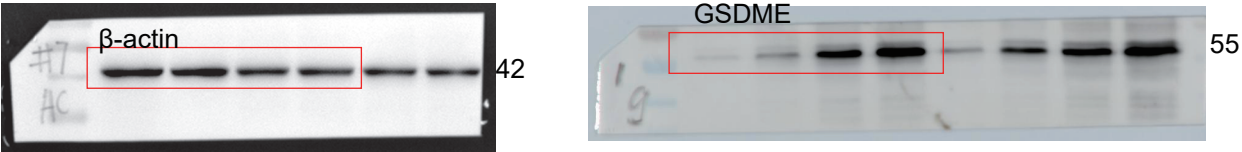

Supplement: Source Data Extended Data Fig. 2 — Unprocessed western blots. [file 41556_2022_857_MOESM16_ESM.pdf]

Figure S3c

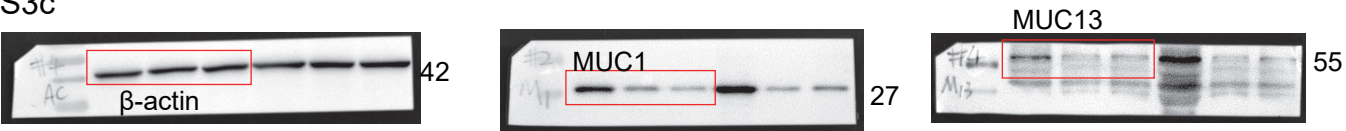

Figure S3d

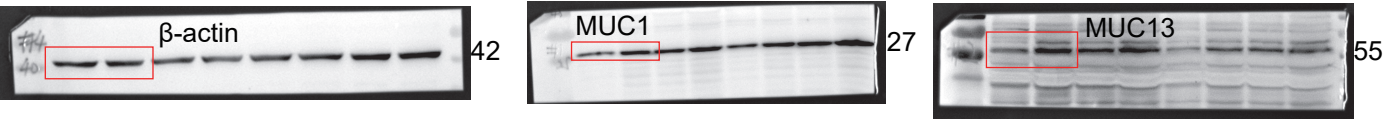

Figure S3e

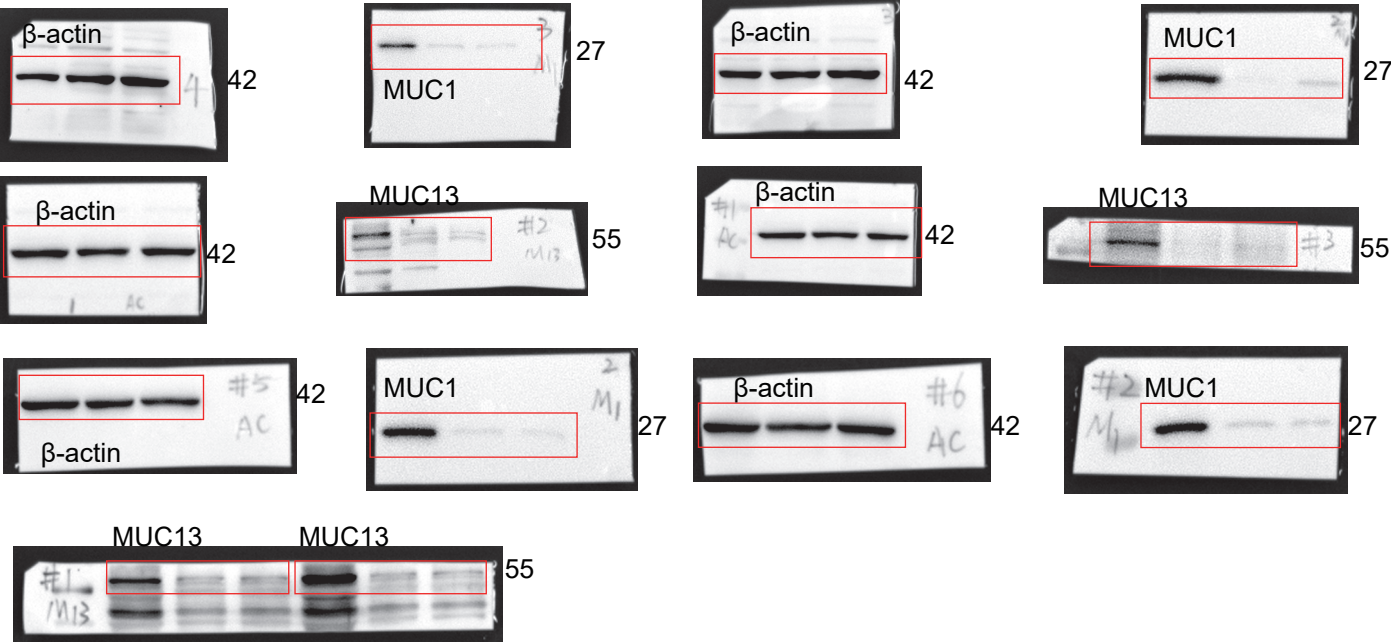

Figure S3k

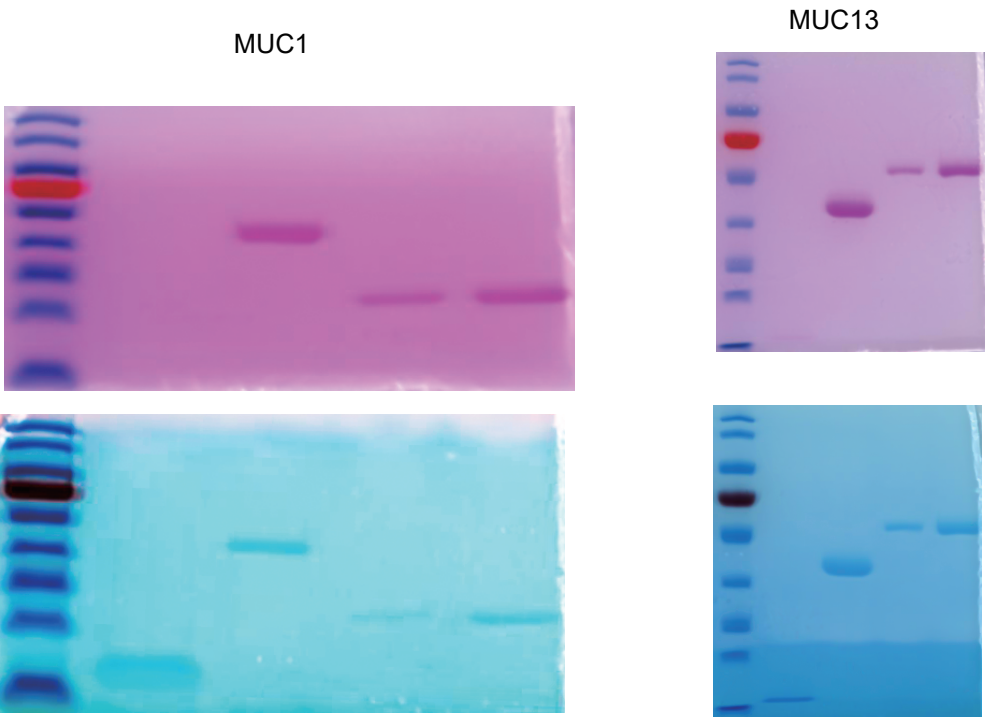

Supplement: Source Data Extended Data Fig. 3 — Unprocessed western blots and gels. [file 41556_2022_857_MOESM18_ESM.pdf]

Figure S4c

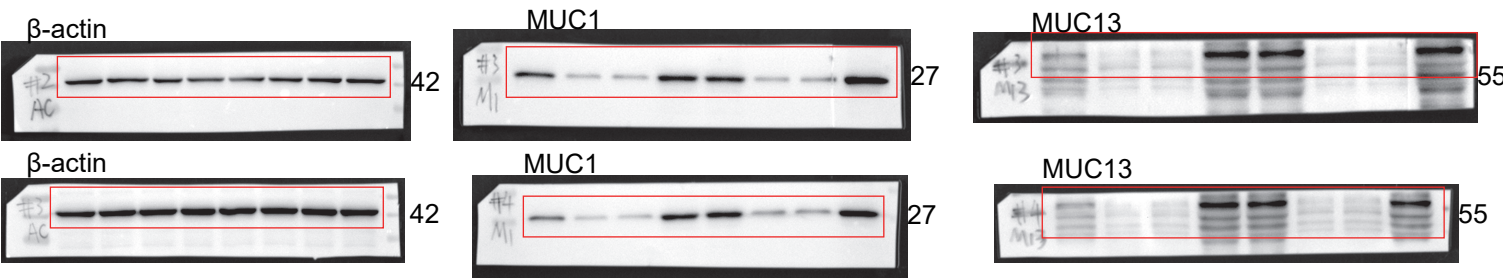

Figure S4e

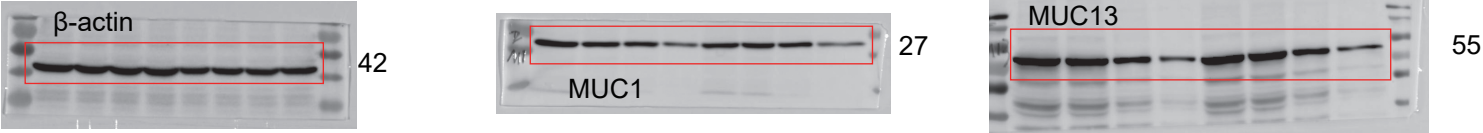

Figure S4j

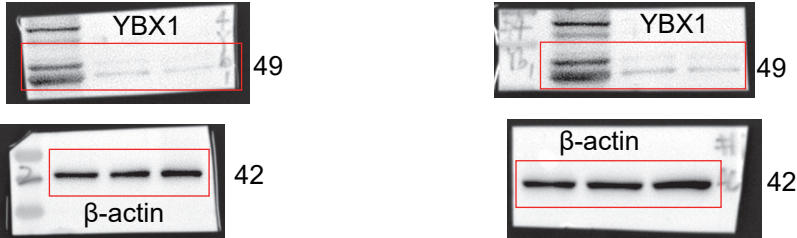

Figure S4l

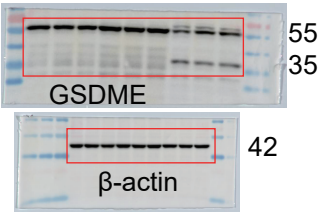

Supplement: Source Data Extended Data Fig. 4 — Unprocessed western blots. [file 41556_2022_857_MOESM20_ESM.pdf]

Figure S5a

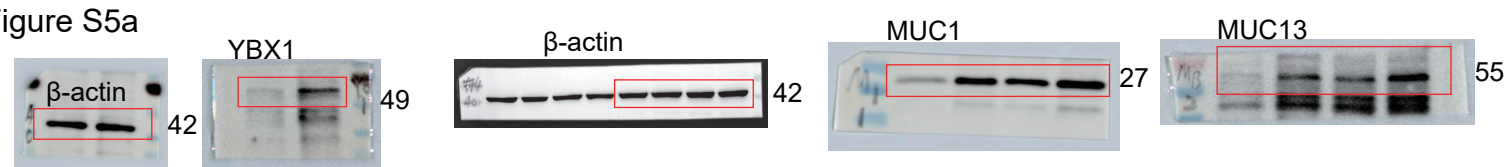

Figure S5b

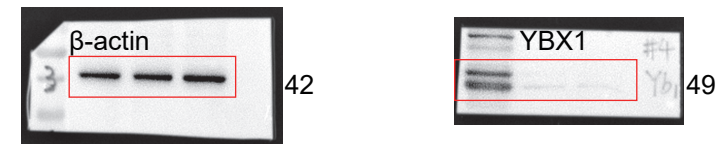

Figure S5e

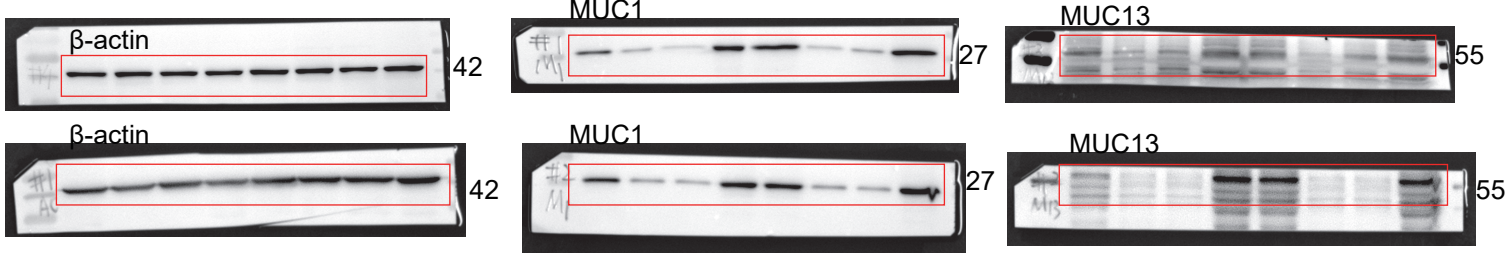

Figure S5g

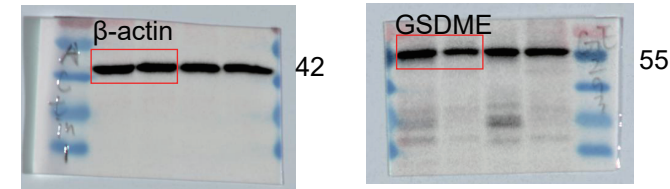

Supplement: Source Data Extended Data Fig. 5 — Unprocessed western blots. [file 41556_2022_857_MOESM22_ESM.pdf]
